# Prospective *in silico* trials identify combined SK and K_2_P channel block as an effective strategy for atrial fibrillation cardioversion

**DOI:** 10.1113/JP287124

**Published:** 2024-11-18

**Authors:** Albert Dasí, Lucas Arantes Berg, Hector Martinez‐Navarro, Alfonso Bueno‐Orovio, Blanca Rodriguez

**Affiliations:** ^1^ Department of Computer Science University of Oxford Oxford UK

**Keywords:** atrial fibrillation, *in silico* trials, K_2_P channels, pharmacological therapy, SK channels, virtual patients

## Abstract

**Abstract:**

Virtual evaluation of medical therapy through human‐based modelling and simulation can accelerate and augment clinical investigations. Treatment of the most common cardiac arrhythmia, atrial fibrillation (AF), requires novel approaches. This study prospectively evaluates and mechanistically explains three novel pharmacological therapies for AF through *in silico* trials, including single and combined SK and K_2_P channel block. AF and pharmacological action were assessed in a large cohort of 1000 virtual patients, through 2962 multiscale simulations. Extensive calibration and validation with experimental and clinical data support their credibility. Sustained AF was observed in 654 virtual patients. In this cohort, cardioversion efficacy increased to 82% (535 of 654) through combined SK+K_2_P channel block, from 33% (213 of 654) and 43% (278 of 654) for single SK and K_2_P blocks, respectively. Drug‐induced prolongation of tissue refractoriness, dependent on the virtual patient's ionic current profile, explained cardioversion efficacy (atrial refractory period increase: 133.0 ± 48.4 ms for combined *vs*. 45.2 ± 43.0 and 71.0 ± 55.3 ms for single SK and K_2_P block, respectively). Virtual patients cardioverted by SK channel block presented lower K_2_P densities, while lower SK densities favoured the success of K_2_P channel inhibition. Both ionic currents had a crucial role on atrial repolarization, and thus a synergism resulted from the multichannel block. All three strategies, including the multichannel block, preserved atrial electrophysiological function (i.e. conduction velocity and calcium transient dynamics) and thus its contractile properties (safety). *In silico* trials identify key factors determining treatment success and the combined SK+K_2_P channel block as a promising strategy for AF management.

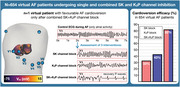

**Key points:**

- This is a large‐scale *in silico* trial study involving 2962 multiscale simulations.
- A population of 1000 virtual patients underwent three treatments for atrial fibrillation.
- Single and combined SK+K_2_P channel block were assessed prospectively.
- The multi‐ion channel inhibition resulted in 82% cardioversion efficacy.
- *In silico* trials have broad implications for precision medicine.

## Graphical abstract

**Abstract figure legend** *In silico* trials were conducted in 654 virtual patients with atrial fibrillation (AF) to assess the cardioversion efficacy of three pharmacological treatments: single SK and K_2_P channel block and combined SK+K_2_P channel inhibition. Left: representative virtual AF patient with the atria inside the torso. Screenshot of the simulated transmembrane voltage and the ECG (dimensionless, only showing atrial activity) in control conditions and after all three pharmacological interventions. Successful AF cardioversion and recovery of P‐waves only occurs for combined SK+K_2_P channel block. Right: cardioversion efficacy in the 654 virtual patients.

## Introduction

Widely accepted in engineering, *in silico* clinical trials now represent a new paradigm in medicine (Viceconti et al., 2021). Similar to clinical trials, *in silico* trials enable the assessment of medical interventions in a patient population, in this case composed of virtual patients using multiscale modelling and simulation. A regulatory framework for *in silico* trials has been proposed (Viceconti et al., 2021), and a growing body of literature is demonstrating their credibility and usefulness (Pappalardo et al., 2019; Passini et al., 2017). *In silico* trials offer a wide range of advantages to augment clinical trials as well as preclinical investigations (Viceconti et al., 2021): simulations can be conducted faster and with a lower economic burden, they are not subjected to ethical constraints, numerous interventions can be applied to the same virtual patient and there is perfect control over the parameters of interest (i.e. less impact of confounding variables).

In this sense, *in silico* trials have proven to be a powerful aid for assessing atrial fibrillation (AF) treatment, the most common cardiac arrhythmia (Dasí et al., 2023). Indeed, a large body of literature has demonstrated their credibility through consistency with experimental and clinical recordings (Bayer et al., 2016; Boyle et al., 2019; Roney et al., 2020). Our recent studies (Dasí et al., 2023, 2024) evidenced the ability of *in silico* trials in large populations of several hundreds of virtual patients to predict and compare retrospectively the efficacy of dozens of AF therapies (pharmacological and ablation). For the first time, we conducted large‐scale *in silico* trials including the patient variability encountered in clinical practice, such as electrophysiological, anatomical and structural factors. This substantially advanced the state‐of‐the‐art compared to previous studies, which considered only a few tens of virtual patients (Bayer et al., 2016; Boyle et al., 2019; Roney et al., 2020), and was crucial to extrapolate *in silico* findings to the interpretation of clinical results. In this sense, the simulations agreed with previously published clinical trials and identified key patient characteristics determining treatment success, which could guide patient stratification to optimal therapies (Dasí et al., 2024). Thus, a further step for *in silico* trials would be predicting the efficacy of interventions yet to be tested in large cohorts of patients (i.e. prospectively) to further support the credibility of these technologies, and to help refine the design of future clinical studies.

Recently, two clinical trials were launched for the assessment of two atrial‐selective targets: the small‐conductance Ca^2+^‐activated K^+^ (SK) and the two‐pore domain K^+^ (K_2_P) channels. Besides being predominantly expressed in the human atria, both the SK current (*I*_SK_) and TASK‐1, a member of the K_2_P current (*I*_K2P_), are up‐regulated in AF patients (Heijman et al., 2023; Kraft et al., 2021). Accordingly, randomized clinical trials were registered to evaluate the cardioversion efficacy of single *I*_SK_ (Holst et al., 2024) and *I*_K2P_ inhibition (DOCTOS trial: EudraCT No. 2018‐002979‐17). The former was stopped prematurely (for Covid‐19‐related slow recruitment) and the results consider a small sample size (Holst et al., 2024). The latter has been recently completed, but the results have yet to be published. Since the available data can only be used to generate hypotheses, further clinical trials with a bigger sample size are needed to determine the real efficacy of either treatment.

Thus, the aim of this study was to conduct a prospective large‐scale *in silico* trial in 1000 virtual patients to (1) assess the AF cardioversion efficacy of three pharmacological interventions (single SK inhibition, K_2_P inhibition and combined SK+K_2_P channel block), and (2) demonstrate the power of *in silico* trials for understanding cardiac arrhythmia mechanisms and selecting appropriate therapies.

## Methods

For the purpose of reproducibility, the bi‐atrial meshes and the configuration files to conduct simulations are publicly available at https://zenodo.org/records/13639663 and /13907120.

### Study population

**Figure 1:**
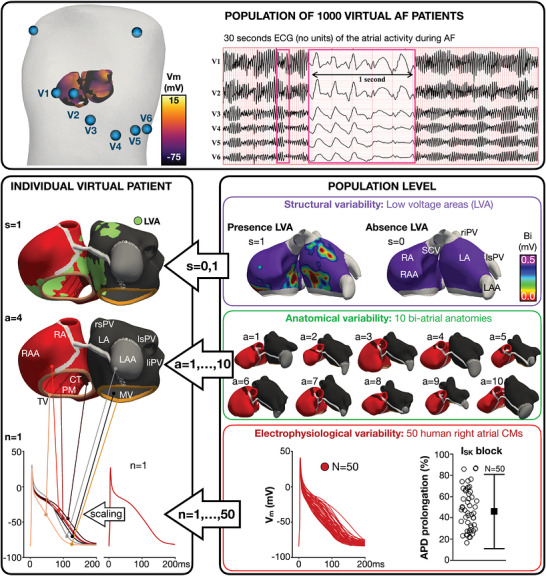
Construction of a population of 1000 virtual patients with atrial fibrillation (AF) *Top*, representative virtual AF patient with the atria inside the torso. Screenshot of the simulated transmembrane voltage and 30 s ECG (dimensionless, only showing atrial activity). *Bottom‐right*, patient characteristics included at the population level: two different structural substrates, absence and presence of LVAs; 10 different bi‐atrial anatomies, with different right and left atrial volumes; and 50 different atrial cardiomyocyte models, with their corresponding action potential duration (APD) prolongation after SK channel block. *Bottom‐left*, construction of a unique virtual patient, including presence of LVAs (s = 1), atrial anatomy number 4 (a = 4) and ionic current profile number 1 (*n* = 1). The latter derives from taking the atrial cardiomyocyte model number 1 (*n* = 1) and scaling it in six other atrial regions. Abbreviations: RA‐LA, right and left atria; RAA‐LAA, RA and LA appendage; TV‐MV, tricuspid and mitral valve; CT, crista terminalis; PM, pectinate muscles; SCV, superior cava vein; rs‐ri‐ls‐li‐PV, right superior, right inferior, left superior and left inferior pulmonary vein.

The construction of a similar cohort is explained in detail elsewhere (Dasí et al., 2024) and briefly summarized here. The 1000 virtual patients were developed based on human data, to cover the variability in structural and electrophysiological substrates commonly encountered in persistent forms of AF (Fig. 1). One half of the cohort was developed without low‐voltage areas (LVAs, bipolar voltage lower than 0.5 mV in sinus rhythm), by combining 50 ionic current profiles and 10 atrial anatomies. These 500 virtual patients were then duplicated into a version that considered LVAs and regions of slow conduction.

We recently demonstrated that the ionic current profile of the atria, rather than the extent of structural remodelling (i.e. LVA infiltration and atrial enlargement), was the key determinant of successful pharmacological therapy (Dasí et al., 2023, 2024). Accordingly, the population of virtual patients was developed using the same bi‐atrial anatomies and the LVA maps described before (Dasí et al., 2024), but with a new population of cardiomyocyte models that better reflected the electrophysiological characteristics of patients with persistent AF in control conditions and after drug treatment.

#### Electrophysiological variability

A population‐of‐models approach was used to capture the ionic current variability commonly observed in experimental data (Britton et al., 2013; Muszkiewicz et al., 2016). The population was constructed using a modified version of the Courtemanche et al. (1998) model, which included the K_2_P channel family, as in Wiedmann et al. (2020), and the SK channel, as in Celotto et al. (2023). Moreover, the maximal conductances of *I*_SK_ and *I*_K2P_ were scaled by 1.1 and 1.7, respectively, to reproduce the 12% and 18% action potential duration (APD) prolongation observed after single SK and K_2_P channel block in cardiomyocytes from control patients (Heijman et al., 2023).

Then, all ionic current densities of the modified model, including the SK and K_2_P channels, were systematically scaled in the range of [0.5, 2.5]. In total, 500 atrial cardiomyocyte models were developed following this process, each of them with a unique combination of ionic current densities. From this population, 50 cardiomyocyte models captured the variability in action potential characteristics of right atrial trabeculae from 149 chronic AF patients (Sanchez et al., 2014) and the APD prolongation after SK channel block observed in right atrial cardiomyocytes from another six AF patients (Heijman et al., 2023; Fig. 1, population level). Each of these 50 models, which reproduced the single‐cell behaviour of right atrial cells from two independent datasets of persistent AF patients, was further scaled to reflect electrophysiological heterogeneities in six atrial regions (Fig. 1, individual virtual patient). The resulting seven action potential models (i.e. the original, representative of the right atrium, and the six scaled ones) configured a unique ionic current profile that was used to populate the atrial anatomies.

#### Anatomical variability

Ten atrial anatomies (with right and left atrial volumes of 127 ± 51 and 105 ± 39 mL, respectively; mean ± SD), spanning the volume ranges observed in persistent AF patients (Lang et al., 2015), were selected. The 500 virtual patients with absence of LVAs derived from the one‐to‐one combination of each atrial anatomy with each ionic profile.

#### Structural variability

These 500 virtual patients were duplicated into a version that included 15% LVAs in the right and left atrium, as this LVA extension is associated with AF recurrence (Dasí et al., 2024). To incorporate LVAs, we used a probabilistic LVA map developed in a previous study. A complete description of the patient data used, the protocols for electro‐anatomical mapping and the development of the probabilistic LVA map can be found in Dasí et al. (2024). Figure 1 shows the 15% bi‐atrial LVA extension on a representative anatomy. LVAs were simulated as regions of 30% decreased conductivity, increased anisotropy (i.e. 8:1 longitudinal to transversal conductivity ratio), and 50, 40 and 50% reductions in *I*_CaL_, *I*_Na_ and *I*_K1_, respectively (Dasí et al., 2023, 2024).

### AF induction and ECG analysis

AF was induced in the population of 1000 virtual patients by imposing spiral wave re‐entries as the initial conditions of the simulation (Dasí et al., 2023, 2024), since the main goal of the study was to assess AF cardioversion and not inducibility. AF lasting longer than 7 s was considered sustained, since we previously showed in a similar cohort (Dasí et al., 2024) that all AF episodes sustaining over 7 s would also sustain for 30 s (i.e. duration used clinically for AF diagnosis). Simulated 12‐lead ECGs were computed in virtual patients with sustained (>7 s) AF, and a representative case with uninterrupted activity for 30 s is illustrated in Fig. 1.

The three‐dimensional monodomain equation of the transmembrane voltage and all ECG calculations were solved using the high‐performance open‐source MonoAlg3D (Sachetto Oliveira et al., 2018).

### Intervention

Virtual patients with sustained (>7 s) AF were independently subjected to three pharmacological interventions: complete inhibition (i.e. ionic current set to zero) of (1) *I*_SK_, (2) *I*_K2P_ and (3) combined *I*_SK_+*I*_K2P_ block. The pharmacological interventions were modelled 2 s from AF initiation and the episode was continued until completion of the original 7 s (i.e. 2 s in the absence of intervention and 5 s after virtual drug administration; Dasí et al., 2023). Longer simulations of 12 s were conducted in a subset of 10 virtual patients to ensure that the cardioversion efficacy and arrhythmia dynamics were independent of the simulation time (results available at https://zenodo.org/records/13907120).

The primary outcome of the study investigated the proportion of virtual patients that were cardioverted before completing the original 7 s (i.e. cardioversion efficacy). The secondary outcome evaluated atrial cardiac safety. Safety regarding ventricular function and proarrhythmia (e.g. QT prolongation) has been already assessed in the clinical trials (Holst et al., 2024). Thus, this investigation focused instead on assessing atrial function (calcium transient and conduction velocity) after *I*_SK_ and *I*_K2P_ block.

The effective refractory period (ERP) was calculated in control conditions and after drug simulation using a cable tissue of 5.00 × 0.04 × 0.04 cm^3^, with a spatial discretization of 400 µm (same as in multiscale simulations). Five consecutive beats were paced at one side of the cable at a constant pacing rate, each time with decreasing cycle lengths. Cardiomyocyte models were previously paced for 50 beats in a single‐cell environment, so they could adapt to the different cycle lengths. The ERP was measured as the shortest cycle length at which all five beats propagated through the cable.

### Statistics

Data normality was assessed by the Kolmogorov–Smirnov test. Non‐parametric data are presented as the median and interquartile range (IQR) and analysed using the Wilcoxon rank sum test. Parametric data are shown as the mean and standard deviation (SD) and analysed through the N‐way analysis of variance. *P* < 0.05 was considered statistically significant.

## Results

### Characteristics of virtual patients with sustained AF

**Figure 2:**
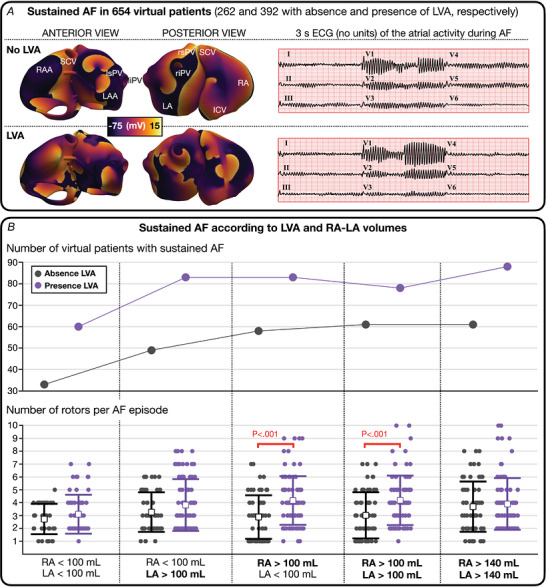
Comparison of atrial fibrillation (AF) dynamics between the subgroup of patients with absence and presence of low voltage areas (LVAs) *A*, screenshot of transmembrane voltage maps and ECG (only atrial activity) of a representative virtual patient with absence (no LVAs) and presence of LVAs. *B*, number of virtual patients with sustained AF and number of rotors per AF episode according to increasing right (RA) and left atrial (LA) volumes (in mL).

From the population of 1000 virtual patients, 654 (65%) presented sustained AF (>7 s). Figure 2*A* illustrates transmembrane voltage maps and corresponding atrial ECGs of two virtual patients, one with absence and one with presence of LVAs. Figure 2*B* shows the number of AF episodes and rotors per episode according to the atrial volume for both subgroups of patients (absence and presence of LVAs).

AF maintenance was favoured by LVAs and larger atria (Fig. 2*B*). LVAs led to more virtual patients sustaining AF (262 *vs*. 392 with absence *vs*. presence of LVAs) and to an increase in AF complexity, qualitatively shown in Fig. 2*A* and quantitatively evidenced by the increase in rotors per AF episode (3.1 ± 1.7 *vs*. 3.9 ± 1.9 with absence *vs*. presence of LVAs; Fig. 2*B*). Similarly, virtual patients with sustained AF were characterized by bigger atrial volumes (44% of virtual patients presented both atrial chambers above 100 mL, 42% at least one chamber over 100 mL and only 14% had both chamber volumes below 100 mL, Fig. 2*B*). Thus, more than half of the cohort with sustained AF (332 of 654) presented LVAs and at least one atrial chamber enlarged.

### Efficacy end‐points

**Figure 3:**
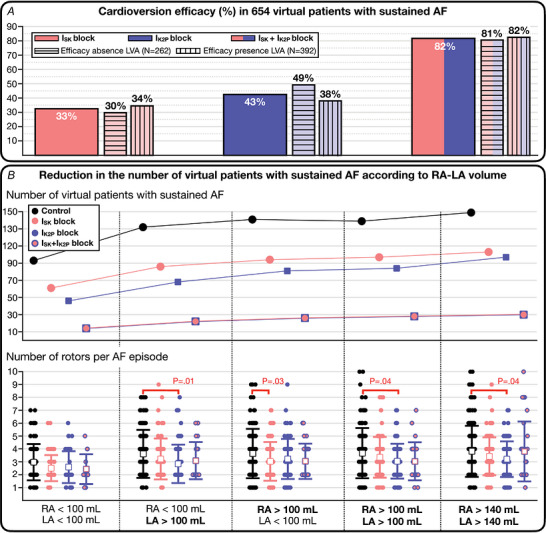
Cardioversion efficacy of single and combined SK and K_2_P channel block *A*, cardioversion efficacy in the 654 virtual patients with sustained atrial fibrillation (AF) (solid bars) and in both subgroups of virtual patients (dashed lines), with presence and absence of low voltage areas (LVAs). *B*, number of virtual patients with sustained AF in control conditions and after application of the three virtual treatments according to increasing right (RA) and left atrial (LA) volumes (in mL). Number of rotors per AF episode in those virtual patients non‐cardioverted by each treatment. The control data represent the 654 virtual patients with sustained AF, considering both absence and presence of LVAs.

The 654 virtual AF patients were subjected to three independent pharmacological treatments, resulting in 2962 multiscale simulations (i.e. 1000 in control conditions and 1962 after drug simulation). Figure 3 illustrates the cardioversion efficacy obtained for the entire population (solid bars) and for both subgroups of virtual patients (dashed bars), with absence (262 of 654) and presence (392 of 654) of LVAs.

Single *I*_SK_ and *I*_K2P_ block exhibited limited cardioversion efficacy, terminating AF in 33% (213 of 654) and 43% (278 of 654) of virtual patients, respectively. Remarkably, combined *I*_SK_+*I*_K2P_ inhibition had a higher cardioversion efficacy than adding the success rate of single channel blocks (i.e. synergism), stopping AF in 535 (82%) cases.

While a similar efficacy derived from single SK and K_2_P channel inhibition, from the 213 virtual patients responding to *I*_SK_ block and the 278 ones responding to *I*_K2P_ block, only 108 virtual patients were cardioverted by both treatments. Conversely, 105 (49%) and 170 (61%) virtual patients cardioverted by single *I*_SK_ and *I*_K2P_ inhibition, respectively, did not respond to the other treatment. Neither strategy showed a strong dependency between cardioversion efficacy and atrial volume (i.e. a similar proportion of virtual patients with different atrial volumes were cardioverted, Fig. 3*B*), and only the efficacy of *I*_K2P_ block was considerably higher in the absence of LVAs (Fig. 3*A*). Furthermore, both treatments, especially *I*_K2P_ block, significantly reduced the number of rotors per AF episode in those virtual patients that were not cardioverted (Fig. 3*B*).

Compared to single channel block, combined *I*_SK_+*I*_K2P_ inhibition yielded a greater reduction in the number of virtual patients with sustained AF (Fig. 3*B*). Indeed, besides cardioverting AF in the majority of cases where single channel block was successful (207 of 213 and 260 of 278 for *I*_SK_ block and *I*_K2P_ inhibition, respectively), the synergistic strategy also terminated AF in 175 additional virtual patients in which neither *I*_SK_ block nor I_K2P_ inhibition worked in isolation.

### Mechanisms of pharmacological cardioversion

**Figure 4:**
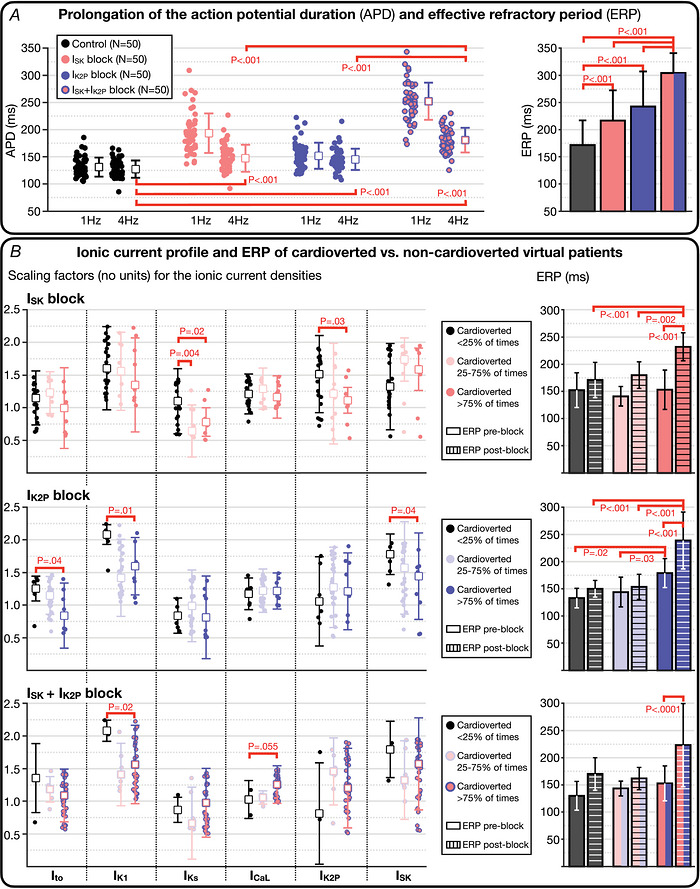
Mechanisms of atrial fibrillation (AF) cardioversion *A*, APD, computed in a single cell at 1 and 4 Hz, and ERP, computed in tissue, of the 50 atrial cardiomyocyte models in control conditions and after applying all three pharmacological treatments. *B‐left*, scaling factors (dimensionless) for the ionic current densities used in ionic profiles that were cardioverted less than 25% of times, between 25 and 75%, and more than 75% of times after applying each pharmacological treatment. *B‐right*, ERP of ionic current profiles that were cardioverted less than 25% of times, between 25 and 75%, and more than 75% after applying each pharmacological treatment. The ERPs are shown before (pre‐block, solid bars) and after (post‐block, dashed bars) applying each strategy.

Figure 4*A* illustrates the drug‐induced prolongation in APD and ERP for all three pharmacological strategies. Longer APDs were observed after combined I_SK_+I_K2P_ block, followed by single *I*_SK_ inhibition and *I*_K2P_ block. When the atrial cardiomyocyte models were paced at 1 Hz, all APD comparisons yielded statistical significance (not included in Fig. 4*A*) and *I*_SK_ inhibition prolonged the APD to a greater extent than *I*_K2P_ block. For 4 Hz pacing, however, no significant difference was obtained between *I*_SK_ block and *I*_K2P_ inhibition, and the combined strategy led to a greater APD prolongation than single channel block (Fig. 4*A*). All three pharmacological treatments significantly prolonged the APD compared to control conditions. At tissue level, the increase in the ERP was proportional to the cardioversion efficacy: higher for combined *I*_SK_+*I*_K2P_ block, followed by *I*_K2P_ inhibition and, lastly, *I*_SK_ block (Fig. 4*A*).

Figure 4*B* compares the ionic current profile and the drug‐induced ERP prolongation between cardioverted and non‐cardioverted virtual patients. Of importance for the analysis, since the same ionic current profile can be used in 20 different virtual patients (i.e. combination of the ionic profile with each of the 10 atrial anatomies, with absence or presence of LVAs), Fig. 4*B* considers three pooled groupings: (1) ionic current profiles rarely cardioverted by the drug [i.e. cardioversion occurred in fewer than 5 (25%) of the 20 virtual patients sharing the same ionic profile]; (2) ionic current profiles usually cardioverted by the drug [i.e. cardioversion occurring in between 5 and 15 (25–75%) virtual patients with the same profile]; and (3) ionic current profiles with frequent cardioversion [i.e. cardioversion in more than 15 (75%) virtual patients with the same ionic profile]. In the text below, virtual patients with ionic current profiles that achieved more than 75% cardioversion are referred to as cardioverted virtual patients, and the remaining virtual patients are considered non‐cardioverted.

Generally, for all three pharmacological strategies, virtual patients showing successful cardioversion also experienced a greater prolongation of refractoriness after the application of the virtual treatment (i.e. ERP post‐block, Fig. 4*B*). For single *I*_SK_ and *I*_K2P_ block, the ERP post‐block of cardioverted patients was not only significantly longer than their corresponding ERP pre‐block but also significantly longer than the ERP post‐block of non‐cardioverted virtual patients. Interestingly, virtual patients cardioverted by *I*_K2P_ block also presented a longer ERP pre‐block than non‐cardioverted virtual patients. This explains that blocking *I*_K2P_ was more efficacious in the absence of LVAs (Fig. 3*A*), in which the ERP was the main determinant of AF maintenance.

The most remarkable finding was that virtual patients frequently cardioverted by *I*_SK_ block presented lower *I*_K2P_ density (alternatively, high *I*_K2P_ hindered the success of *I*_SK_ block), and similarly, *I*_K2P_ inhibition was successful in virtual patients with lower *I*_SK_ density (alternatively, high *I*_SK_ hampered *I*_K2P_ block). This finding suggests that, since both ionic currents had an important role on tissue refractoriness, blocking one current was not effective when the other one was up‐regulated. Moreover, it explains the high efficacy of the synergistic block, remaining only unsuccessful in virtual patients with the shortest refractoriness (due to concomitant *I*_K1_ up‐regulation and *I*_CaL_ down‐regulation, see Fig. 4*B*).

Additionally, *I*_SK_ block was less efficacious in virtual patients with *I*_Ks_ up‐regulation and with a non‐significant tendency for lower *I*_SK_. On the other hand, *I*_K2P_ inhibition had a lower efficacy in virtual patients with increased *I*_to_ and *I*_K1_ (Fig. 4*B*). As expected, the cardioversion efficacy of single channel block was less efficacious in virtual patients with a strong repolarization reserve.

### Safety end‐points

**Figure 5:**
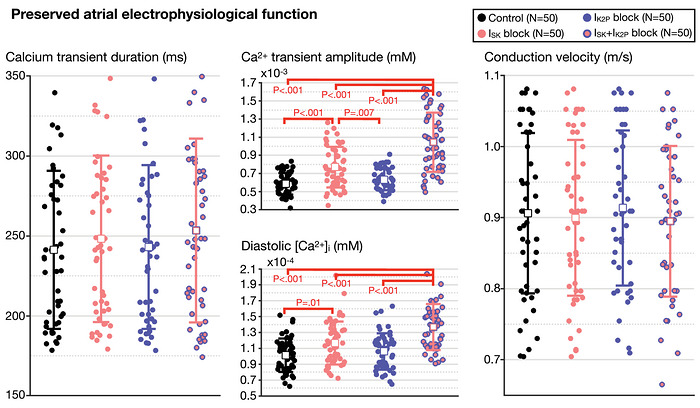
Safety end‐points of SK and K_2_P channel block Comparison of calcium transient biomarkers and longitudinal conduction velocity in the bulk atrial tissue between control conditions and after virtual application of single and combined SK and K_2_P channel inhibition.

Neither pharmacological treatment produced a prolongation of the calcium transient duration or a reduction in the longitudinal conduction velocity compared to control conditions. Only the calcium transient amplitude and the diastolic level were slightly higher after SK channel block and thus also after combined *I*_SK_+*I*_K2P_ inhibition (Fig. 5).

## Discussion

This is a prospective, large‐scale *in silico* trial study considering 1000 virtual patients to evaluate single and combined application of two new AF therapies (SK and K_2_P channel inhibition). *In silico* trials exploit the complete control and transparency of modelling and simulation to identify key factors determining response to different therapies in the same cohort of virtual patients. Our results reveal that AF cardioversion efficacy was proportional to an increase in tissue refractoriness determined by the specific pharmacological therapy and the underlying patient's ionic profile. When considering single channel block, *I*_K2P_ and *I*_SK_ up‐regulation had a crucial role on the success of pharmacological treatment, which emphasized the benefits of adopting a multi‐ion channel block strategy.

### Comparison to preclinical and clinical studies

We previously conducted *in silico* trials in a similar population of 800 virtual patients to assess optimal pharmacological and catheter ablation therapy for AF (Dasí et al., 2024). The credibility of the *in silico* trials was supported by consistency with experimental and clinical data, and also with the efficacy reported for 12 therapies in their corresponding clinical trials and studies (Dasí et al., 2024).

For the current study, we used the same methodological framework as in our extensively validated work, but developed a larger population of 1000 virtual patients to include the single‐cell effects of SK channel block observed in atrial cardiomyocytes from persistent AF patients (Heijman et al., 2023). Thus, compared to the 33% (213 of 654) of virtual patients cardioverted *in silico*, AF cardioversion rates of 43% (5 of 12) and 55% (12 of 22) have been reported in a phase‐2 clinical trial of AP30663 (SK channel blocker) at 3 and 5 mg/kg, respectively (Holst et al., 2024). The higher cardioversion efficacy observed in human patients might be due to many reasons, for instance the small sample size of the trial, the fact that only paroxysmal AF patients were enrolled, or that besides inhibiting the SK channel, AP30663 also produces an off‐target inhibition of Kv11.1 (hERG gene) (Holst et al., 2024).

Interestingly, some studies have proposed a direct or indirect inhibition of *I*_Na_ by SK channel blockers, which could also explain the lower efficacy *in silico*. Experiments in paced goats observed that AP14145 produced a reduction of the conduction velocity (Gatta et al., 2021). Moreover, NS8593 and ICAGEN decreased the action potential amplitude and maximum upstroke velocity in tissue from patients in sinus rhythm and AF (Skibsbye et al., 2014). Experiments in dogs even proposed that NS8593 had no effect on the APD, but prolonged the atrial ERP (Burashnikov et al., 2020). Since the latter agent significantly reduced *I*_Na_ in HEK cells, the authors suggested a main anti‐arrhythmic effect based on the inhibition of the sodium channel.

However, these observations have not been replicated in other or even the same studies. Gatta et al. (2021) did not observe a similar reduction in the conduction velocity during AF, proving a lack of rate‐dependent inhibition of *I*_Na_ by AP14145. Similarly, earlier work in pigs also demonstrated that AP14145 did not produce a significant effect on *I*_Na_. Indeed, there are several studies confirming the lack of affinity of most SK channel blockers, AP14145 (Dines et al., 2017), NS8593 and ICAGEN (Skybsbye et al., 2014) and AP30663 (Bentzen et al., 2020), for *I*_Na_.

The most compelling evidence is presented by *in vivo* studies reporting the effect of AP30663 in humans (Gal et al., 2020; Yfanti et al., 2024). Both studies demonstrated that the SK channel blocker AP30663 did not produce a significant effect on the P‐wave duration. This is strong evidence supporting that AP30663, the reference compound cited in the present study, has no effect on atrial conduction velocity, at least at normal frequencies, which validates the results presented in Fig. 5.

That being said, the *in silico I*_SK_ block reproduced an ideal scenario, where complete SK channel inhibition was achieved. In a study conducted in pigs (Diness et al., 2020), 5 mg/kg of AP30663 produced a free plasma concentration equivalent to the IC_50_ of the SK channels (i.e. 50% inhibition of *I*_SK_). While a higher cardioversion efficacy could have been obtained in the clinical trial if a higher dose (e.g. 25 mg/kg) had been tested (Holst et al., 2024), an administration of 8 mg/kg of AP30663 was associated with a high‐risk QT interval prolongation in healthy volunteers (Yfanti et al., 2024). In pigs (Diness et al., 2020), a dose of 25 mg/kg increased the atrial ERP by 71% (+83 ± 7 ms). This is in agreement with the ERP prolongation (+73.6 ± 47.4 ms) seen in the current study for cardioverted virtual patients. Therefore, future studies should attempt to synthesize agents that produce a complete SK channel block without yielding an off‐target inhibition of ionic currents such as *I*_Kr_ that increase the risk of QT interval prolongation.

To the best of our understanding, the atrial ERP modulation by SK channel block has not been reported in humans, but we ensured that our *in silico* cardiomyocyte models reproduced the APD prolongation observed in patients with AF (Heijman et al., 2023).

Regarding *I*_K2P_ inhibition, the results from the DOCTOS trial are yet to be published. In animal studies, a 100% cardioversion rate was observed in 17 pigs that received 1.8 mg/kg doxapram (a TASK‐1 inhibitor; Wiedmann et al., 2022). At a similar dose (2.0 mg/kg), the porcine atrial refractory period was prolonged on average by 38.3, 25.8 and 42.5 ms at cycle lengths of 500, 400 and 300 ms, respectively (Wiedmann et al., 2022). In our simulations, a further ERP prolongation of 71.0 ± 55.3 ms was observed in human virtual patients. In isolated cardiomyocytes from persistent AF patients, *I*_K2P_ inhibition increased the APD up to 30% (Kraft et al., 2021), compared to an average and a maximum prolongation of 16 ± 8 and 34.4%, respectively, observed in our simulations. Thus, we anticipate that the DOCTOS trial will report a comparable efficacy in AF patients as that observed *in silico* after K_2_P channel block.

The *in silico* results captured the ERP prolongation observed in several animal models after SK and K_2_P block, supporting the credibility of the simulations. Human studies assessing ERP changes, as well as bigger clinical trials with SK and K_2_P channel blockers are needed to further validate our findings.

### Comparison to previous simulation studies

A study by Ni et al. (2020) hypothesized, using cable tissues, that the combined block of several atrial‐selective K^+^ currents would provide a synergistic anti‐arrhythmic effect. The authors assessed the impact of single and multiple channel block on the APD, ERP and conduction velocity in 1D settings. We simulated AF in anatomical atrial meshes (i.e. realistic 3D organ representations) and, in agreement with Ni et al (2020), a greater ERP prolongation was observed for the combined *I*_SK_+*I*_K2P_ block, followed by single inhibition of *I*_K2P_ and, lastly, *I*_SK_ block. Furthermore, the authors observed a negligible effect on the conduction velocity after blocking these channels, also shown in the current study (Fig. 5).

Another and more recent study by the same group (Herrera et al., 2023) observed that under AF electrophysiological remodelling, *I*_SK_, *I*_K2P_ and *I*_K1_ were the K^+^ currents with the most prominent role in the atrial ERP. Consistent with their single‐cell results, we observed in realistic atrial meshes that *I*_SK_ and *I*_K2P_ up‐regulation dictated the success of pharmacological cardioversion, and when both ionic currents were inhibited, cardioversion only failed in those virtual patients with *I*_K1_ up‐regulation. Furthermore, the authors observed that increased SK channel density reduced the amplitude of the calcium transient, or equivalently as we observed, increased calcium transient amplitude derived from *I*_SK_ block (Fig. 5).

As in the present study, both previous works (Herrera et al., 2023; Ni et al., 2020) used a population of cardiomyocyte models approach to include electrophysiological variability. Importantly, the authors employed a different cellular model to build the population, which demonstrates model independence of key results.

### Efficacy and safety benefits of multitargeting atrial‐selective ionic currents

With the completion of preliminary trials assessing *I*_SK_ block (Holst et al., 2024) and *I*_K2P_ inhibition (DOCTOS), all atrial‐selective ionic currents, including the acetylcholine‐dependent K^+^ current (*I*_K,ACh_; Podd et al., 2016) and the ultra‐rapid rectifier K^+^ current (*I*_Kur_; Camm et al., 2019), have been evaluated for rhythm control of AF in randomized clinical trials.

*I*_Kur_ was considered a very attractive target since the inhibition of this major repolarization current in the human atria was expected to prolong the ERP and destabilize rotors. The DIAGRAF‐IKUR trial, however, ended prematurely, showing no meaningful reduction in disease burden in paroxysmal AF patients (Camm et al., 2019). Several reasons were given to explain the limited success of *I*_Kur_ block; for example, some studies demonstrated a decreased *I*_Kur_ density in cardiomyocytes from AF patients (Dobrev & Ravens, 2003).

This was not the case for *I*_K,ACh_ (also *I*_SK_ and *I*_K2P_), activity of which appeared consistently up‐regulated in AF patients (Dobrev & Ravens, 2003). Yet, the trial assessing *I*_K,ACh_ inhibition also reported a lack of efficacy for reducing AF burden (Podd et al., 2016). The authors speculated that the compound used might have not blocked *I*_K,ACh_ in AF patients. A more interesting reason, also highlighted by the investigators of the DIAGRAF‐IKUR trial, was that meaningful AF prevention needed more than the single inhibition of one atrial‐selective current.

In agreement with the latest statement, we have demonstrated that targeting multiple atrial‐selective ionic currents results in a synergistic cardioversion efficacy (i.e. greater than adding the individual efficacy of single channel block). Moreover, the benefits of multiple channel block could also be extended to cardiac safety. For example, ventricular cardiomyocytes from heart failure patients show *I*_SK_ up‐regulation (extensively reviewed by Herrera et al., 2023). Thus, *I*_SK_ inhibition might be associated with safety concerns in AF patients with concomitant heart failure. Moreover, both doxapram (*I*_K2P_ inhibitor) and AP30663 (SK channel blocker) produce an off‐target effect on the hERG current at high doses. Thus, a lower‐dose, multitarget strategy would potentially be more efficacious and safer, but clinical evidence, especially concerning ventricular safety associated with the combined strategy, is needed to demonstrate this statement.

### Sex‐specific response to SK and K2P channel inhibition

The population of 1000 virtual patients was developed and calibrated considering at all stages both male and female data. In this sense, the atrial cardiomyocyte models were compared to action potential traces from 149 persistent AF patients, from which 37% were female. The bi‐atrial anatomies were developed using 47 (30% female) clinical computed tomography and magnetic resonance imaging datasets, and the bi‐atrial electro‐anatomical maps of 20 (41% female) AF patients were used to incorporate LVAs in the generated anatomies.

However, a sub‐analysis of the cardioversion efficacy considering male *vs*. female virtual patients was not performed. A recent experimental study identified that ventricular cardiomyocytes from female rabbits showed higher SK current density than male animals, yielding a greater reduction of the APD under increased sympathetic drive (Chen et al., 2018). A higher *I*_SK_ in females would have both cardiac efficacy and safety implications. In our study, K_2_P channel inhibition was less effective under *I*_SK_ up‐regulation. Therefore, if the results observed in rabbit ventricles were present in the human atria, K_2_P channel block would be more effective in men than in women, and vice versa for SK channel inhibition. At the same time, however, *I*_SK_ block could result in a greater QT prolongation and ventricular proarrhythmia in women (Herrera et al., 2023), given the increased SK channel density in female ventricular cardiomyocytes.

Thus, considering sex‐specific differences could be critical for planning pharmacological treatment. However, human data, especially from the atria, are needed to confirm sex differences in SK and K_2_P channels.

### Limitations

This study aimed to study AF cardioversion in a large population of virtual patients. Thus, a compromise was reached between the total number of simulations (i.e. 2962) and the duration of each simulation (i.e. 7 s). However, it is difficult to anticipate whether 7 s of simulated AF is equivalent to the 90 min waiting reported in the trials (Holst et al., 2024) and thus if it could be used to predict AF cardioversion. In this sense, since pharmacokinetics (i.e. time course of drug absorption and bioavailability in the organisms) are not included in the drug modelling, the simulated interventions produce an immediate effect. Thus, *in silico* trials can predict within a very short time whether a drug might be effective in terminating an arrhythmia. Likewise, since human modelling and simulation allows perfect control over the parameters of interest, a mechanistic explanation can be provided for treatment success. Figure 4 illustrates that cardioverted virtual patients experienced a significant prolongation of the ERP after drug application, which failed to happen in those patients not cardioverted. Accordingly, non‐cardioverted virtual patients presented stable rotors in the atria even after drug application, which remained unmodified during the entire duration of the AF episode (i.e. similar number and location of rotors over time). The significant difference in ERP prolongation between cardioverted *vs*. non‐cardioverted virtual patients and the stable AF dynamics seen in non‐cardioverted patients after simulated drug treatment is reassuring that longer simulation times would be unlikely to change the outcomes of the study. Indeed, in a representative cohort of 10 virtual patients with variability in anatomy and electrophysiology that were not cardioverted after 5 s of simulated drug action, a longer simulation time of 10 s did not result in AF cardioversion or in a reduction of arrhythmia complexity. These simulations are available at https://zenodo.org/records/13907120.

Furthermore, the simulations reproduced ideal pharmacological conditions, in which a complete SK and K_2_P channel inhibition was explored. While this might not be feasible in clinical practice, we have ensured the APD and ERP prolongation *in silico* match the results obtained in experimental and clinical conditions. Moreover, if strong evidence is presented regarding the effect of SK channel blockers on *I*_Na_, future studies may expand the present investigations evaluating such a contribution on conduction velocity and tissue excitability.

Lastly, while combined SK+K_2_P channel block seems an effective strategy for AF cardioversion, a greater clinical challenge is maintaining sinus rhythm. Thus, further studies should attempt to evaluate whether the combined pharmacological approach is suitable for long‐term rhythm control of AF.

## Conclusion

Our prospective, large‐scale *in silico* trial identifies key factors determining the success of combined *versus* single SK and K_2_P channel block, highlighting the polypharmacological inhibition as a safe and very effective strategy for AF management. Moreover, this study strengthens the power of *in silico* trials based on human modelling and simulation for understanding optimal cardiac therapies.

## Additional information

## Competing interests

The authors declare that they have no competing interests.

## Author contributions

All authors contributed equally to the conception of the work and revising it critically for important intellectual content, and gave their final approval of the version to be published, ensuring that questions related to the accuracy or integrity of any part of the work were appropriately investigated and resolved. A.D. was responsible for conducting all the simulations, analysis of results and drafting the work. B.R. and A.B.‐O. acted as supervisors of the research. L.A.‐B. and H.M.‐N. mainly contributed to methodological aspects.

## Funding

This work received funding from the EPSRC Impact Acceleration Account Award (UKRI Grant Reference ‐ EP/X525777/1) (to A.D.). The project was also supported by a Wellcome Trust Senior Fellowship in Basic Biomedical Sciences (214 290/Z/18/Z to B.R.), the CompBioMed and CompBiomed2 Centre of Excellence in Computational Biomedicine (European Union's Horizon 2020; grant agreement 675 451 and 823 712), and the CompBiomedX EPSRC‐funded project (EP/X019446/1). We acknowledge additional support from the Oxford BHF Centre of Research Excellence (RE/13/1/30 181).

## Supporting information


Peer Review History

## Data Availability

The bi‐atrial meshes and the configuration files to conduct simulations are publicly available at https://zenodo.org/records/13639663 and https://zenodo.org/records/13907120. Further inquiries can be directed to the corresponding authors.
