# Supplementary material for: Prospective in silico trials identify combined SK and K2P channel block as an effective strategy for atrial fibrillation cardioversion
Source: J Physiol. 2024 Nov 18;604(13):5221–35. doi: 10.1113/JP287124 (PMC13327755; doi:10.1113/JP287124)
Supplement: Supplementary file 1 — Peer Review History [file TJP-604-5221-s001.pdf]

The Journal of Physiology

Prospective in-silico trials identify combined SK and K2P channel block as an effective strategy for atrial fibrillation cardioversion

Albert Dasí, Lucas Arantes Berg, Hector Martinez-Navarro, Alfonso Bueno Orovio, and Blanca Rodriguez
DOI: 10.1113/JP287124

Corresponding author(s): Albert Dasí (albert.dasiimartinez@cs.ox.ac.uk)

The referees have opted to remain anonymous.

Review Timeline:

Submission Date:	18-Jun-2024
Editorial Decision:	21-Aug-2024
Revision Received:	18-Sep-2024
Editorial Decision:	03-Oct-2024
Revision Received:	14-Oct-2024
Accepted:	23-Oct-2024

Senior Editor: Bjorn Knollmann

Reviewing Editor: Viviane Timmermann

Transaction Report:

(Note: With the exception of the correction of typographical or spelling errors that could be a source of ambiguity, letters and reports are not edited. Depending on transfer agreements, referee reports obtained elsewhere may or may not be included in this compilation. Referee reports are anonymous unless the Referee chooses to sign their reports.)

Dear Dr Dasí,

Re: JP-RP-2024-287124 "Prospective in-silico trials identify combined SK and K2P channel block as an effective strategy for atrial fibrillation cardioversion" by Albert Dasí, Lucas Arantes Berg, Hector Martinez-Navarro, Alfonso Bueno Orovio, and Blanca Rodriguez

Thank you for submitting your manuscript to The Journal of Physiology. It has been assessed by a Reviewing Editor and by 2 expert referees and we are pleased to tell you that it is potentially acceptable for publication following satisfactory major revision.

Please advise your co-authors of this decision as soon as possible.

The referee reports are copied at the end of this email.

Please address all the points raised and incorporate all requested revisions or explain in your Response to Referees why a change has not been made. We hope you will find the comments helpful and that you will be able to return your revised manuscript within 2 months. If you require longer than this, please contact journal staff: jp@physoc.org. Please note that this letter does not constitute a guarantee for acceptance of your revised manuscript.

Your revised manuscript should be submitted online using the link in your Author Tasks: Link Not Available. This link is accessible via your account as Corresponding Author; it is not available to your co-authors. If this presents a problem, please contact journal staff (jp@physoc.org). Image files from the previous version are retained on the system. Please ensure you replace or remove any files that are being revised.

If you do not wish to submit a revised version of your manuscript, you must inform our journal staff (jp@physoc.org) or reply to this email to request withdrawal. Please note that a manuscript must be formally withdrawn from the peer review process at one journal before it may be submitted to another journal.

TRANSPARENT PEER REVIEW POLICY: To improve the transparency of its peer review process, The Journal of Physiology publishes online as supporting information the peer review history of all articles accepted for publication. Readers will have access to decision letters, including Editors' comments and referee reports, for each version of the manuscript, as well as any author responses to peer review comments. Referees can decide whether or not they wish to be named on the peer review history document.

ABSTRACT FIGURES: Authors are expected to use The Journal's premium BioRender account to create/redraw their Abstract Figures. Information on how to access this account is here: https://physoc.onlinelibrary.wiley.com/journal/14697793/biorender-access.

This will enable Authors to create and download high-resolution figures. If authors have used the free BioRender service, they can use the instructions provided in the link above to download a high-resolution version suitable for publication.

The link provided should only be used for the purposes of this submission. Authors will be charged for figures created on this account if they are not related to this manuscript submission.

LANGUAGE EDITING AND SUPPORT FOR PUBLICATION: If you would like help with English language editing, or other article preparation support, Wiley Editing Services offers expert help, including English Language Editing, as well as translation, manuscript formatting, and figure formatting at www.wileyauthors.com/eeo/preparation. You can also find resources for Preparing Your Article for general guidance about writing and preparing your manuscript at www.wileyauthors.com/eeo/prepresources.

RIGOUR AND REPRODUCIBILITY: We recommend authors consult The Journal's Rigour and Reproducibility requirements to ensure that their manuscript is suitable for publication: https://physoc.onlinelibrary.wiley.com/pb-assets/hub-assets/physoc/documents/TJP-Rigour-and-Reproducibility-Requirements.pdf

REVISION CHECKLIST:

Check that your Methods section conforms to journal policy: https://jp.msubmit.net/cgi-bin/main.plex?form_type=display_requirements#methods.

Check that data presented conforms to the statistics policy: https://jp.msubmit.net/cgi-bin/main.plex?form_type=display_requirements#statistics.

Upload a full Response to Referees file. To create your 'Response to Referees': copy all the reports, including any comments from the Senior and Reviewing Editors, into a Microsoft Word, or similar, file and respond to each point, using font or background colour to distinguish comments and responses and upload as the required file type.

Please upload two versions of your manuscript text: one with all relevant changes highlighted and one clean version with no changes tracked. The manuscript file should include all tables and figure legends, but each figure/graph should be uploaded as separate, high-resolution files.

You may also upload:

- 'Potential Cover Art' for consideration as the issue's cover image
- Appropriate Supporting Information (Video, audio or data set: see https://jp.msubmit.net/cgi-bin/main.plex?form_type=display_requirements#supp).

We look forward to receiving your revised submission.

If you have any queries, please reply to this email and we will be pleased to advise.

Yours sincerely,

Bjorn Knollmann
Senior Editor
The Journal of Physiology

REQUIRED ITEMS

- Include a Key Points list in the article itself, before the Abstract.

- Author photo and profile. First or joint first authors are asked to provide a short biography (no more than 100 words for one author or 150 words in total for joint first authors) and a portrait photograph. These should be uploaded and clearly labelled together in a Word document with the revised version of the manuscript. See Information for Authors for further details.

- You must start the Methods section with a paragraph headed Ethical Approval. If experiments were conducted on humans, confirmation that informed consent was obtained, preferably in writing, that the studies conformed to the standards set by the latest revision of the Declaration of Helsinki and that the procedures were approved by a properly constituted ethics committee, which should be named, must be included in the article file. If the research study was registered (clause 35 of the Declaration of Helsinki), the registration database should be indicated, otherwise the lack of registration should be noted as an exception (e.g. The study conformed to the standards set by the Declaration of Helsinki, except for registration in a database). For further information see: https://physoc.onlinelibrary.wiley.com/hub/human-experiments.

- The reference list must be in alphabetical order, rather than numbered, to comply with our Journal format.

- Your manuscript must include a complete Additional Information section, including competing interests; funding; author contributions and acknowledgements.

- A Data Availability Statement is required for all papers reporting original data. This must be in the Additional Information section of the manuscript itself. It must have the paragraph heading 'Data Availability Statement'. All data supporting the results in the paper must be either: in the paper itself; uploaded as Supporting Information for Online Publication; or archived in an appropriate public repository. The statement needs to describe the availability or the absence of shared data. Authors must include in their statement: a link to the repository they have used, or a statement that it is available as Supporting Information; reference the data in the appropriate sections(s) of their manuscript; and cite the data they have shared in the References section. Whenever possible, the scripts and other artefacts used to generate the analyses presented in the paper should also be publicly archived. If sharing data compromises ethical standards or legal requirements then authors are not expected to share it, but must note this in their statement. For more information, see our Statistics Policy.

- Please include an Abstract Figure file, as well as the Figure Legend text within the main article file. The Abstract Figure is a piece of artwork designed to give readers an immediate understanding of the research and should summarise the main

conclusions. If possible, the image should be easily 'readable' from left to right or top to bottom. It should show the physiological relevance of the manuscript so readers can assess the importance and content of its findings. Abstract Figures should not merely recapitulate other figures in the manuscript. Please try to keep the diagram as simple as possible and without superfluous information that may distract from the main conclusion(s). Abstract Figures must be provided by authors no later than the revised manuscript stage and should be uploaded as a separate file during online submission labelled as File Type 'Abstract Figure'. Please also ensure that you include the figure legend in the main article file. All Abstract Figures should be created using BioRender. Authors should use The Journal's premium BioRender account to export high-resolution images. Details on how to use and access the premium account are included as part of this email.

EDITOR COMMENTS

Reviewing Editor:

I agree with the referees' comments but would like to add that it is crucial for the journal that the results are reproducible. Therefore, it would be important to make the code accessible to other scientists, as the link to Zenodo (l. 74) requires permission and is currently not operational.

Please also see 'Required Items' above.

Senior Editor:

I concur with the assessment by the reviewing editor. The work is of interest to the Journal, but a responsive revision requires addressing the reviewers concerns and making the code is accessible to other scientists.

REFEREE COMMENTS

Referee #1:

The manuscript by Dasí et al. presents a computational study that investigates the efficacy of SK and K2P channel inhibition, and of their combination, to terminate atrial fibrillation (AF). The authors use 1000 virtual models of human atria that include different atrial anatomies and electrophysiological and structural properties, and they test the efficacy of SK and K2P channel inhibition in 654 out of these 1000 models that show "sustained" AF. They conclude that the synergistic effect of the combined channel block represents a promising treatment therapy, and is more potent than inhibition of SK or K2P channel alone.

The antiarrhythmic effects of SK and K2P channel inhibition have been previously described in both experimental and clinical settings, and the presented results on single SK and K2P channel block principally correspond to the previously published studies. The novelty of the presented study therefore lies in predicting the potential synergistic effect of combined SK and K2P channel inhibition that could be indeed an interesting atrial-selective approach for pharmacological treatment of AF. The study uses previously established and validated computational models. The manuscript is well-written and the results are clearly presented.

Major comments and suggestions:

1) I find the major limitation of the study in modeling the SK channel inhibition. SK channel block has been shown to be associated with decreased tissue excitability, and this effect has been reported for several compounds, including NS8593 and ICAGEN (Skybsbye et al. 2014), AP14145 (Gatta et al. 2021), and even for AP30663 (Yfanti et al. 2024) that the authors mention in their manuscript. The decreased excitability is likely caused by depolarization of the resting membrane potential, and it has been proposed that the antiarrhythmic effect of SK channel inhibition results from the synergy of class III effect (direct SK channel block) and class I effect (reduction of sodium currents) (Skybsbye et al. 2014, Gatta et al. 2021). Figure 5 shows no effect of SK channel inhibition on conduction velocity, indicating that the modeling approach the authors applied in their study does not take this aspect of SK channel inhibition in account.

The absence of reduced sodium current could explain the relatively low efficacy of simulated SK channel inhibition to terminate AF when compared to the clinical trial with AP30663 (33% vs. 43% or 55%, lines 285-286). I find the decreased

tissue excitability as an important aspect of SK channel inhibition, and I recommend the authors to address it in their work. In my opinion, the study would greatly benefit from additional simulations evaluating the combined effect of SK channel block and reduced sodium current.

2) The sentence "Thus, an even higher cardioversion efficacy could have been obtained in the clinical trial if a higher dose (i.e. 25 mg/kg) had been tested." (lines 293-295) gives an impression that using high doses of the SK channel blocker AP30663 could be feasible in patients. However, this is not true. As reported by Yfanti et al., administration of 8 mg/kg of AP30663 was associated with the QTcF interval prolongation that represented too high risk for the volunteers. I think the authors should clearly mention in Discussion that a complete SK channel block, and possibly a complete K2P block, are likely not feasible, and the cardioversion efficacy of the combined SK and K2P channel block reported in the study is an upper estimate of the cardioversion efficacy such treatment could possibly have.

Minor comments and suggestions

3) The study reports on "Safety endpoints", stating that "Neither pharmacological treatment produced a detrimental change on atrial electrophysiological function" (lines 260-261). Figure 5 then shows several parameters, with significant changes in Ca^{2+} transient amplitude and Diastolic Ca^{2+}. What were the safety limits for each of the parameters in Figure 5?

4) As far as I understand, the control data in Figure 3B represent the sum of the data with presence and absence of LVA from Figure 2B. Is that correct? If so, I suggest to mention this in the description of Figure 3B.

5) The title of Figure 2A misses "respectively".

References

Skibsbye et al., Cardiovascular Research (2014), DOI: 10.1093/cvr/cvu121

Gatta et al., Europace (2021), DOI: 10.1093/europace/euab125

Yfanti et al., British Journal of Clinical Pharmacology (2024), DOI: 10.1111/bcp.15973

Referee #2:

In this work, A. Dasí et al developed an in-silico trial study that targeted three different strategies for AF management including i) single SK channel block, ii) single K2P channel block, and iii) the combination of both. The computational results suggest that the combined application of both channel blockers provides a better solution. Although the study is limited to numerical analyses and does not include a validation of the hypotheses in vivo, they offer an exciting strategy that may motivate future clinical work. Overall, the work is interesting and the research topic is well aligned with the journal scope. However, there are some issues and limitations that have to be addressed before publication:

- In the abstract, the authors stated that the computational model has been calibrated and validated with experimental and clinical data. This statement should be better supported in the manuscript. Although the calibration strategy is well detailed, it is not clear how the calibrated model is validated.

- The authors introduced a new population of cardiomyocyte models that better reflected the electrophysiological characteristics of patients. The specific changes in these models with respect to previous work should be clarified.

- The distribution of low voltage areas is a key feature in the simulations. The authors should better describe the LVA

locations and the motivation/clinical source for them. Is it based on MRI human data?

- In line 101 the authors used a population of model approach to capture the experimental variability. Please include the reference.

- The authors scale the maximal conductances by 1.1 and 1.7 to reproduce the APD prolongation observed in control patients. Then, in the discussion the authors highlighted that the model reproduced this APD prolongation. It is not clear if this is an in silico outcome that supports the model validation or if this is only an imposition by the authors. Please discussed in more detail.

-Please specify the formulation used for ISK. Does this include a function describing the calcium dependent activation as the one optimised in [https://doi.org/10.1161/CIRCRESAHA.122.321858]?

- How many models were filtered with the first database (AP morphology) and how many with the second (APD prolongation after ISK block)?

- Please include the reference for line 121 (volume rage observed in persistent AF patients).

- I am concerned with the statement in line 135: "AF lasting longer than 7 s was considered sustained, since we previously showed in a similar cohort that all AF episodes sustaining over 7 s would also sustain for 30 s (i.e., duration used clinically for AF diagnosis)". I am not sure about this statement for the cases with smaller atrial dimensions. Can the authors demonstrate this and provide examples for the extreme scenarios?

- Line 384: the authors state that "non-cardioverted virtual patients presented stable rotors in the atria even after drug application, which remained unmodified during the entire duration of the AF episode". Could the authors be more specific (i.e., is the dominant frequency checked over time, number of rotors, etc)?

Minor comments: The sentence in line 187 is repetitive with the previous paragraph.

END OF COMMENTS

EDITOR COMMENTS

Reviewing Editor

I agree with the referees' comments but would like to add that it is crucial for the journal that the results are reproducible. Therefore, it would be important to make the code accessible to other scientists, as the link to Zenodo (l. 74) requires permission and is currently not operational.

We appreciate the time and effort from the editors and reviewers. The manuscript has been revised considering the reviewers' comments. A point-by-point response to those comments is provided below. Moreover, the link to ZENODO has been fixed and it is now operational.

New link: https://zenodo.org/records/13639663.

Please also see 'Required Items':

- **Include a Key Points list in the article itself, before the Abstract.**

  Added

- **Author photo and profile. First or joint first authors are asked to provide a short biography (no more than 100 words for one author or 150 words in total for joint first authors) and a portrait photograph. These should be uploaded and clearly labelled together in a Word document with the revised version of the manuscript. See Information for Authors for further details.**

  The author profile and picture are attached to the revised version.

- **You must start the Methods section with a paragraph headed Ethical Approval. If experiments were conducted on humans, confirmation that informed consent was obtained, preferably in writing, that the studies conformed to the standards set by the latest revision of the Declaration of Helsinki and that the procedures were approved by a properly constituted ethics committee, which should be named, must be included in the article file. If the research study was registered (clause 35 of the Declaration of Helsinki), the registration database should be indicated, otherwise the lack of registration should be noted as an exception (e.g., The study conformed to the standards set by the Declaration of Helsinki, except for registration in a database).**

  Not applicable. This is a simulation study.

- **The reference list must be in alphabetical order, rather than numbered, to comply with our Journal format.**

  The reference list has been changed to alphabetical order.

- **Your manuscript must include a complete Additional Information section, including competing interests; funding; author contributions and acknowledgements.**

  This information is included in the main article file.

- **A Data Availability Statement is required for all papers reporting original data. This must be in the Additional Information section of the manuscript itself. It must have the paragraph heading 'Data Availability Statement'. All data supporting the results in the paper must be either: in the paper itself; uploaded as Supporting Information for Online Publication; or archived in an appropriate public repository. The statement needs to describe the availability or the absence of shared data. Authors must include in their statement: a link to the repository they have used, or a statement that it is available as Supporting Information; reference the data in the appropriate sections(s) of their manuscript; and cite the data they have shared in the References section. Whenever possible, the scripts and other artefacts used to generate the analyses presented in the paper should also be publicly archived. If sharing data compromises ethical standards or legal requirements then authors are not expected to share it, but must note this in their statement. For more information, see our Statistics Policy.**

  A Data Availability Statement has been added at the end of the manuscript.

- **Please include an Abstract Figure file, as well as the Figure Legend text within the main article file. The Abstract Figure is a piece of artwork designed to give readers an immediate understanding of the research**

and should summarise the main conclusions. If possible, the image should be easily 'readable' from left to right or top to bottom. It should show the physiological relevance of the manuscript so readers can assess the importance and content of its findings. Abstract Figures should not merely recapitulate other figures in the manuscript. Please try to keep the diagram as simple as possible and without superfluous information that may distract from the main conclusion(s). Abstract Figures must be provided by authors no later than the revised manuscript stage and should be uploaded as a separate file during online submission labelled as File Type 'Abstract Figure'. Please also ensure that you include the figure legend in the main article file.

The Abstract Figure is uploaded separately and its legend is included in the main article file.

Senior Editor

I concur with the assessment by the reviewing editor. The work is of interest to the Journal, but a responsive revision requires addressing the reviewers concerns and making the code is accessible to other scientists.

We agree with the Senior Editor. The manuscript has been revised considering the reviewers' comments and a point-by-point response to those comments is provided below. The data and tools used to conduct this study are now publicly available.

REFEREE COMMENTS

Referee #1:

The manuscript by Dasí et al. presents a computational study that investigates the efficacy of SK and K2P channel inhibition, and of their combination, to terminate atrial fibrillation (AF). The authors use 1000 virtual models of human atria that include different atrial anatomies and electrophysiological and structural properties, and they test the efficacy of SK and K2P channel inhibition in 654 out of these 1000 models that show "sustained" AF. They conclude that the synergistic effect of the combined channel block represents a promising treatment therapy, and is more potent than inhibition of SK or K2P channel alone.

The antiarrhythmic effects of SK and K2P channel inhibition have been previously described in both experimental and clinical settings, and the presented results on single SK and K2P channel block principally correspond to the previously published studies. The novelty of the presented study therefore lies in predicting the potential synergistic effect of combined SK and K2P channel inhibition that could be indeed an interesting atrial-selective approach for pharmacological treatment of AF. The study uses previously established and validated computational models. The manuscript is well-written and the results are clearly presented.

We thank the reviewer for their positive comments. In addition to the mentioned strengths, we would still like to emphasize that the advancements and novelties of this work extend beyond our findings on therapy efficacy, and include novel methods for studying it. From a technological standalone point-of-view, the present study represents one of the largest multi-scale computational studies to date on therapy evaluation, including 2,962 multi-scale simulations of atrial fibrillation and pharmacological treatment in a population of 1,000 virtual patients. This substantially surpasses the state-of-the-art in human computer modelling and simulation compared to previous efforts, which for example simulated drug testing in 25 virtual patients (doi:10.3389/fphys.2021.650449) or ablation in 100 virtual patients (doi:10.1161/CIRCEP.121.010253). For the first time, we enable large-scale in-silico clinical trials exhibiting patient variability as encountered in clinical practice, including electrophysiological, anatomical, and structural factors. This is crucial to extrapolate in-silico findings to the design and interpretation of clinical trials for many cardiovascular diseases.

Major comments and suggestions:

1. **I find the major limitation of the study in modeling the SK channel inhibition. SK channel block has been shown to be associated with decreased tissue excitability, and this effect has been reported for several compounds, including NS8593 and ICAGEN (Skybsbye et al. 2014), AP14145 (Gatta et al. 2021), and even for AP30663 (Yfanti et al. 2024) that the authors mention in their manuscript. The decreased excitability is likely caused by depolarization of the resting membrane potential, and it has been proposed that the antiarrhythmic effect of SK channel inhibition results from the synergy of class III effect (direct SK channel block) and class I effect (reduction of sodium currents) (Skybsbye et al. 2014, Gatta et al. 2021). Figure 5 shows no effect of SK channel inhibition on conduction velocity, indicating that the modeling approach the authors applied in their study does not take this aspect of SK channel inhibition in account.**

   The reviewer rises an interesting point for discussion: whether SK channel block exerts a pure class III effect or a combined class III and class I action.

   Experiments in paced goats observed that the SK channel blocker AP14145 produced a reduction of the conduction velocity (Gata et al., 2021). Moreover, other agents, such as NS8593 and ICAGEN, yielded a reduction in the action potential amplitude and maximum upstroke velocity in tissue from patients in sinus rhythm and AF (Skibsbye et al., 2014). Experiments in dogs even proposed that NS8593 had no effect on the APD, but prolonged the atrial ERP (Burashnikov et al., 2020). The latter study showed that NS8593 significantly reduced I_{Na} in HEK cells. Therefore, we acknowledge that there are some observations that point to a direct or indirect inhibition of I_{Na} by SK channel blockers.

   However, these observations are weakly supported. Gata *et al.*, 2021 did not observe a similar reduction in the conduction velocity during AF, proving that AP1414 does not possess the rate-dependence

property of class Ic agents. Moreover, the QRS also remained unchanged after applying AP1414, evidencing no ventricular reduction in conduction velocity. Ultimately, the authors acknowledged the impossibility of unravelling the mechanism (if any) behind a possible class I effect. Earlier work in pigs also demonstrated that AP14145 did not produce a significant effect on I_{Na} or any other current apart from I_{SK}, I_{Kr} and $I_{K,ACh}$ (Dines et al., 2017).

Indeed, several other studies have shown no significant effect on I_{Na} for most SK channel blockers: AP14145 (Dines et al., 2017), NS8593 and ICAGEN (Skybsbye et al. 2014), and AP30663 (Bentzen et al., 2020). Perhaps the most compelling evidence is presented by the *in vivo* studies reporting the administration of AP30663 in humans (Gal et al., 2020; Yfanti et al., 2023). Both studies demonstrated that the **SK channel blocker AP30663 did not produce a significant effect on the P-wave duration.** This is a strong proof supporting that AP30663, the reference compound that we cite in our paper, has no effect on atrial conduction velocity, at least at normal frequencies, in agreement with our **Figure 5**.

Altogether, given the lack of strong evidence supporting it, we decided to not **explicitly** inhibit I_{Na} in our modelling approach. Given that a reduction in conduction velocity has only been observed in goats and for one single compound and that there is plenty of evidence, in humans, supporting normal conduction after SK channel block, we believe that the modelling approach adopted in our study for SK channel block is accurate. Moreover, as indicated in the Discussion, we are modelling an ideal scenario where SK channel block is purely selective and does not affect any other ionic current.

This explanation has been added in the Discussion:

Line 311 – 329: *Interestingly, some studies have proposed a direct or indirect inhibition of I_{Na} by SK channel blockers, which could also explain the lower efficacy in-silico. Experiments in paced goats observed that AP14145 produced a reduction of the conduction velocity (Gata et al., 2021). Moreover, NS8593 and ICAGEN decreased the action potential amplitude and maximum upstroke velocity in tissue from patients in sinus rhythm and AF (Skibsbye et al., 2014). Experiments in dogs even proposed that NS8593 had no effect on the APD, but prolonged the atrial ERP (Burashnikov et al., 2020). Since the latter agent significantly reduced I_{Na} in HEK cells, the authors suggested a main antiarrhythmic effect based on the inhibition of the sodium channel.*

However, these observations have not been replicated in other or even the same studies. Gata et al., did not observe a similar reduction in the conduction velocity during AF, proving a lack of rate-dependent I_{Na} inhibition by AP1414. Similarly, earlier work in pigs also demonstrated that AP14145 did not produce a significant effect on I_{Na}. Indeed, there are several studies confirming the lack of affinity of most SK channel blockers, AP14145 (Dines et al., 2017), NS8593 and ICAGEN (Skybsbye et al., 2014) and AP30663 (Bentzen et al., 2020), for I_{Na}.

The most compelling evidence is presented by the in vivo studies reporting the effect of AP30663 in humans (Gal et al., 2020; Yfanti et al., 2023). Both studies demonstrated that the SK channel blocker AP30663 did not produce a significant effect on the P-wave duration. This is a strong proof supporting that AP30663, the reference compound cited in the present study, has no effect on atrial conduction velocity, at least at normal frequencies, which validates the results presented in ***Figure 5****.*

2. **The absence of reduced sodium current could explain the relatively low efficacy of simulated SK channel inhibition to terminate AF when compared to the clinical trial with AP30663 (33% vs. 43% or 55%, lines**

285-286). I find the decreased tissue excitability as an important aspect of SK channel inhibition, and I recommend the authors to address it in their work. In my opinion, the study would greatly benefit from additional simulations evaluating the combined effect of SK channel block and reduced sodium current.

As mentioned in the Discussion, there are plenty of procedural differences supporting the lower efficacy *in silico* compared to the cardioversion efficacy observed in human patients, for instance the small sample size of the clinical trial (i.e., 34 patients) compared to our 654 virtual patients, the fact that only paroxysmal AF patients were enrolled in the clinical study while we simulated persistent AF, or that, besides inhibiting the SK channel, AP30663 also produces an off-target inhibition of Kv11.1 (I_{Kr}).

Moreover, we indeed show decreased tissue excitability after SK channel block and reported an ERP prolongation of 45.2±43.0 after I_{SK} inhibition.

As mentioned before, there is no evidence in humans supporting a **direct inhibition** of I_{Na} by AP30663 or any other SK channel blocker. Therefore, if we were to simulate a combined effect of SK channel block and reduced sodium current, it would be unclear to know what representative percentage of block should we consider for the latter, leading to methodological uncertainty. In addition, we have also previously shown (10.3389/fphys.2022.966046; 10.1113/JP284730) that a weak inhibition of I_{Na} (e.g., 30% block) might have pro-arrhythmic potential, since it reduces the conduction velocity without producing a sufficient prolongation of post-repolarization refractoriness, favouring the formation of stable rotors.

Nevertheless, we have added a final line in the discussion saying that shall strong evidence be presented about the effect of SK channel blockers on I_{Na}, future studies may expand the present investigations evaluating such a contribution on conduction velocity:

Line 446 – 448: *Moreover, shall strong evidence be presented about the effect of SK channel blockers on I_{Na}, future studies may expand the present investigations evaluating such a contribution on conduction velocity and tissue excitability.*

3. **The sentence "Thus, an even higher cardioversion efficacy could have been obtained in the clinical trial if a higher dose (i.e. 25 mg/kg) had been tested." (lines 293-295) gives an impression that using high doses of the SK channel blocker AP30663 could be feasible in patients. However, this is not true. As reported by Yfanti et al., administration of 8 mg/kg of AP30663 was associated with the QTcF interval prolongation that represented too high risk for the volunteers. I think the authors should clearly mention in Discussion that a complete SK channel block, and possibly a complete K2P block, are likely not feasible, and the cardioversion efficacy of the combined SK and K2P channel block reported in the study is an upper estimate of the cardioversion efficacy such treatment could possibly have.**

We thank the reviewer for their comment. We have modified the explanation in the Discussion to reflect the reviewer's remark:

Line 330 – 340: *The in-silico I_{SK} block reproduced an ideal scenario, where a complete SK channel inhibition was achieved. In a study conducted in pigs (Diness et al., 2020), 5 mg/kg of AP30663 produced a free plasma concentration equivalent to the IC_{50} of the SK channels (i.e., 50% inhibition of I_{SK}). While a higher cardioversion efficacy could have been obtained in the clinical trial if a higher dose (e.g., 25 mg/kg) had been tested (Holst et al., 2023), an administration of 8 mg/kg of AP30663 was associated with a high-risk QT interval prolongation in healthy volunteers (Yfanti et al., 2023). In pigs (Diness et al., 2020), a dose of 25 mg/kg increased by 71% (+83±7 ms) the atrial ERP. This is in agreement with the ERP prolongation (+73.6±47.4 ms) seen in the current study for cardioverted*

virtual patients. Therefore, future studies should attempt to synthesize agents that produce a complete SK channel block without yielding an off-target inhibition of ionic currents such as I_{Kr}, that increase the risk of QT interval prolongation.

Moreover, the fact that a complete SK and K_2P channel block might not be feasible in the clinic has been acknowledged in the Limitations:

Line 443 – 446: *The simulations reproduced ideal pharmacological conditions, in which a complete SK and K_2P channel inhibition was explored. While this might not be feasible in clinical practice, we have ensured the APD and ERP prolongation in-silico match the results obtained in experimental and clinical conditions.*

Minor comments and suggestions

1. **The study reports on "Safety endpoints", stating that "Neither pharmacological treatment produced a detrimental change on atrial electrophysiological function" (lines 260-261). Figure 5 then shows several parameters, with significant changes in Ca2+ transient amplitude and Diastolic Ca2+. What were the safety limits for each of the parameters in Figure 5?**

   We thank the reviewer for their comment. We agree that the original sentence was vague and somehow inaccurate. We have rewritten the section regarding "Safety endpoints":

   Line 280 – 283: *Neither pharmacological treatment produced a prolongation of the calcium transient duration or a reduction in the longitudinal conduction velocity compared to control conditions. Only the calcium transient amplitude and the diastolic level were slightly higher after SK channel block and thus, also after combined I_{SK}+I_{K2P} inhibition (Figure 5).*

2. **As far as I understand, the control data in Figure 3B represent the sum of the data with presence and absence of LVA from Figure 2B. Is that correct? If so, I suggest to mention this in the description of Figure 3B.**

   The reviewer is correct, this description has been added in the legend of Figure 3B.

   Text in legend of Figure 3B: *The control data represent the 654 virtual patients with sustained AF, considering both absence and presence of LVA.*

3. **The title of Figure 2A misses "respectively".**

   Added.

Referee #2:

In this work, A. Dasí et al developed an in-silico trial study that targeted three different strategies for AF management including i) single SK channel block, ii) single K2P channel block, and iii) the combination of both. The computational results suggest that the combined application of both channel blockers provides a better solution. Although the study is limited to numerical analyses and does not include a validation of the hypotheses in vivo, they offer an exciting strategy that may motivate future clinical work. Overall, the work is interesting and the research topic is well aligned with the journal scope. However, there are some issues and limitations that have to be addressed before publication:

1. **In the abstract, the authors stated that the computational model has been calibrated and validated with experimental and clinical data. This statement should be better supported in the manuscript. Although the calibration strategy is well detailed, it is not clear how the calibrated model is validated.**

   We thank the reviewer for their comment. At tissue level, the atrial cardiomyocyte models reproduced the ERP prolongation observed for several animal models after SK and K2P channel block. This was the first validation of the multi-scale simulations.

   Moreover, similar primary endpoints were obtained after SK channel block in the clinical trial (i.e., cardioversion efficacy of 43-55%) and the in-silico trial (33%), despite several procedural differences (e.g., small sample size of the trial, enrolling 34 patients compared to our 654 virtual patients, the fact that only paroxysmal AF patients were enrolled while we simulated persistent AF, or that, besides inhibiting the SK channel, AP30663 also produces an off-target inhibition of I_{Kr}).

   Likewise, our computational framework was based on an extensively validated study, in which we reproduced the primary endpoint of clinical trials and studies for 12 AF therapies, including catheter ablation and pharmacological treatment.

   Lastly, similar to in vivo studies, we reported no effect on conduction properties after SK channel inhibition.

   This has been emphasized in the main text:

   Line 302 – 304: *For the current study, we used the same methodological framework as in our extensively validated work, but developed a larger population of 1000 virtual patients to include the single-cell effects of SK channel block observed in atrial cardiomyocytes from persistent AF patients (Heijman et al., 2023).*

   Line 325 – 329: *The most compelling evidence is presented by the in vivo studies reporting the effect of AP30663 in humans (Gal et al., 2020; Yfanti et al., 2023). Both studies demonstrated that the SK channel blocker AP30663 did not produce a significant effect on the P-wave duration. This is a strong proof supporting that AP30663, the reference compound cited in the present study, has no effect on atrial conduction velocity, at least at normal frequencies, which validates the results presented in Figure 5.*

   Line 354 – 357: *The in-silico results captured the ERP prolongation observed in several animal models after SK and K_2P block, supporting the credibility of the simulations. Human studies assessing ERP changes, as well as bigger clinical trials with SK and K_2P channel blockers are needed to further validate our findings.*

2. **The authors introduced a new population of cardiomyocyte models that better reflected the electrophysiological characteristics of patients. The specific changes in these models with respect to previous work should be clarified.**

The specific changes applied to the baseline Courtemanche cellular model to develop the population of 50 atrial cardiomyocyte models are detailed in **Table S1**. These values can be found in the configuration files uploaded to ZENODO (publicly available at https://zenodo.org/records/13639663).

Table S1. Scaling factors used for developing the in-silico population of human atrial cardiomyocyte (aCM) models.

**aCM model**	**Scaling factors (no units) applied to the ionic current densities of the Courtemanche model**										
	G_{Kur}	G_{Kr}	G_{to}	G_{K1}	G_{Ks}	G_{CaL}	G_{NaK}	G_{NCX}	G_{Na}	G_{K2P}	G_{SK}
**aCM model 1**	1.40	0.71	0.63	1.98	1.14	1.41	0.66	0.83	0.72	1.9	1.04
**aCM model 2**	0.53	1.30	1.03	0.79	0.91	1.15	1.03	0.56	0.78	0.68	1.59
**aCM model 3**	1.06	0.77	0.88	1.52	0.57	1.08	1.13	0.70	1.10	0.66	1.94
**aCM model 4**	0.56	1.19	1.17	1.53	0.60	1.01	1.44	0.91	0.67	1.64	1.67
**aCM model 5**	1.17	0.98	0.89	1.68	0.98	1.32	1.05	0.73	0.64	1.83	1.83
**aCM model 6**	0.58	0.54	1.24	1.27	0.73	1.11	1.09	0.52	0.87	0.53	1.57
**aCM model 7**	0.90	0.60	1.25	1.26	0.84	1.06	0.70	0.66	1.38	0.69	1.20
**aCM model 8**	0.76	1.48	0.60	1.15	0.79	1.4	0.82	0.68	1.32	1.57	1.95
**aCM model 9**	1.07	1.09	0.77	1.42	1.38	1.07	0.70	0.58	0.96	0.82	1.15
**aCM model 10**	0.79	0.67	1.42	2.14	0.72	1.26	0.65	1.24	0.83	0.91	1.52
**aCM model 11**	0.89	0.55	1.37	1.84	0.83	1.44	0.61	1.44	1.46	0.89	1.87
**aCM model 12**	0.98	1.33	1.15	1.54	1.01	1.36	1.26	1.19	0.95	1.77	1.88
**aCM model 13**	0.82	0.97	0.65	1.41	1.10	1.11	0.84	0.80	0.96	1.68	1.17
**aCM model 14**	1.43	0.69	1.24	2.10	0.51	1.34	0.54	1.45	1.40	0.74	1.58
**aCM model 15**	1.49	1.10	0.90	0.93	1.19	1.39	0.52	0.59	1.00	0.70	1.05
**aCM model 16**	0.92	0.97	1.36	2.25	0.87	1.17	1.18	0.54	1.03	1.75	1.80
**aCM model 17**	0.54	0.94	1.47	1.15	0.74	1.10	1.21	0.53	0.79	1.19	1.00
**aCM model 18**	1.13	0.50	1.38	2.08	0.84	1.03	0.69	0.89	0.64	0.72	1.32
**aCM model 19**	1.38	1.45	1.23	1.98	0.97	1.45	0.65	1.13	1.25	1.15	1.78
**aCM model 20**	1.24	0.52	1.16	1.69	0.59	0.92	0.95	1.07	1.15	1.51	1.81
**aCM model 21**	0.90	0.64	1.00	1.87	0.83	1.02	0.90	1.36	1.43	1.03	1.83
**aCM model 22**	1.28	1.05	0.99	1.36	0.99	1.21	1.03	0.73	0.54	1.72	1.90
**aCM model 23**	1.07	0.87	1.27	1.90	1.4	1.24	0.86	0.87	1.46	1.19	1.10
**aCM model 24**	1.40	0.78	0.84	1.59	1.37	1.49	0.71	0.69	1.45	1.65	0.56
**aCM model 25**	0.88	0.54	0.55	2.25	0.78	1.33	0.52	1.02	0.60	1.29	0.73
**aCM model 26**	0.65	1.43	1.36	1.21	0.99	1.30	1.32	0.64	0.71	0.84	1.36
**aCM model 27**	1.44	0.60	1.23	1.05	0.68	0.98	1.45	0.57	0.66	1.27	1.94
**aCM model 28**	1.22	0.74	1.18	1.80	1.39	1.46	0.92	0.51	1.47	1.25	0.89
**aCM model 29**	0.77	0.74	1.05	1.86	1.43	1.40	1.14	1.07	1.05	1.88	1.69
**aCM model 30**	1.19	0.56	0.83	1.71	0.51	1.33	0.98	0.88	1.29	0.91	0.85
**aCM model 31**	0.52	0.68	1.24	2.07	0.59	1.07	1.06	0.73	0.57	1.40	1.24
**aCM model 32**	1.16	0.74	0.81	1.11	1.18	0.89	0.67	0.56	1.01	0.77	1.94
**aCM model 33**	0.56	1.17	0.79	1.30	1.12	1.05	0.78	0.96	0.78	1.11	1.89
**aCM model 34**	1.34	1.42	0.57	1.03	0.51	0.99	0.56	0.62	0.76	1.21	1.51
**aCM model 35**	1.42	0.65	1.40	1.11	0.81	1.25	0.66	0.78	0.85	1.47	0.55
**aCM model 36**	1.36	0.62	0.68	2.02	1.1	0.79	0.70	0.64	0.63	0.81	1.89
**aCM model 37**	0.76	1.03	1.21	1.21	1.14	1.04	1.02	0.58	1.31	1.70	1.41
**aCM model 38**	0.51	0.50	0.98	2.22	0.77	1.47	0.71	1.18	1.43	1.12	1.63
**aCM model 39**	1.35	1.18	1.41	1.59	0.56	1.39	0.97	0.66	0.65	0.51	0.63
**aCM model 40**	1.41	1.15	0.71	1.24	0.94	0.94	0.52	0.51	1.49	0.59	1.65
**aCM model 41**	1.37	1.42	1.09	1.36	0.55	1.13	0.63	0.84	0.93	0.85	1.85
**aCM model 42**	1.42	1.05	1.47	1.18	0.60	1.33	0.92	0.68	1.40	1.36	1.06
**aCM model 43**	1.16	1.31	0.85	1.12	1.14	1.41	1.23	0.72	1.15	1.55	1.32
**aCM model 44**	0.63	0.66	0.58	1.77	0.52	1.25	0.57	0.61	0.58	0.67	0.74
**aCM model 45**	1.47	1.01	1.25	2.14	0.66	1.24	0.50	1.11	0.90	1.06	1.95
**aCM model 46**	1.15	0.83	1.41	1.96	1.27	1.43	0.76	1.40	0.67	1.62	1.70
**aCM model 47**	1.32	0.87	1.09	1.39	1.27	1.22	0.79	0.81	0.94	1.01	0.84
**aCM model 48**	0.78	0.53	0.62	1.66	0.61	1.00	0.74	1.47	1.12	1.14	1.60
**aCM model 49**	1.31	0.69	0.94	1.60	1.25	1.22	0.55	1.01	0.77	1.16	1.44
**aCM model 50**	0.62	1.21	1.05	1.30	1.36	0.97	1.38	0.80	0.77	1.79	1.21

3. **The distribution of low voltage areas is a key feature in the simulations. The authors should better describe the LVA locations and the motivation/clinical source for them. Is it based on MRI human data?**

We appreciate the reviewer's comment. The LVA were based on electro-anatomical maps of 20 (76% persistent, 41% female) AF patients, which were obtained at the John Radcliffe Hospital in Oxford.

High density mapping of the atria was performed using an Abbott Advisor HD grid (SE) catheter while pacing at the coronary sinus at a cycle length of 800 ms. The patient electro-anatomical maps were registered to the endocardial surface of a bi-atrial statistical shape model, through a rigid and non-rigid registration. Then, the bipolar voltage data of all 20 patients were superimposed into the same atrial geometry to build a probabilistic LVA map, i.e., identifying those atrial regions most likely to undergo LVA remodelling. These most probable areas were assigned to be LVA until 15% of the left and right atrium was remodelled. Finally, the LVA distribution created in the bi-atrial statistical shape model was extended to the 10 atrial anatomies.

Therefore, the location of LVA is based on the most probable location found in 20 patients with AF. As can be seen in **Figure 1**, LVA usually infiltrate in the anterior wall of the left and right atrium. Some LVA also appear in the posterior and inferior wall of the left atrium, and the lateral wall of the right atrium.

This is explained in detail in our previous publication, and we have now clarified it in the main article:

Line 144 – 146: *To incorporate LVA, we used a probabilistic LVA map developed in a previous study. A complete description of the patient data used, the protocols for electro-anatomic mapping and the development of the probabilistic LVA map can be found in Dasí et al., 2024.*

4. **In line 101 the authors used a population of model approach to capture the experimental variability. Please include the reference.**

A reference has been added next to population of model approach:

Line 119 – 120: *A population of models approach was used to capture the ionic current variability commonly observed in experimental data (Britton et al., 2013; Muszkiewicz et al., 2016).*

5. **The authors scale the maximal conductances by 1.1 and 1.7 to reproduce the APD prolongation observed in control patients. Then, in the discussion the authors highlighted that the model reproduced this APD prolongation. It is not clear if this is an in-silico outcome that supports the model validation or if this is only an imposition by the authors. Please discussed in more detail.**

We thank the reviewer's comment. We scaled by 1.1 and 1.7 the maximal conductance of SK and K2P channels, respectively, to reproduce the APD prolongation observed in patients in sinus rhythm (i.e., control patients). Moreover, during calibration, we ensured that the APD prolongation reproduced by the models was consistent with that observed in **patients with AF** after SK channel block.

The reviewer is right when saying that both conditions were imposed during the calibration of the models, and this has been clarified in the discussion:

Line 341 – 343: *To the best of our understanding, the atrial ERP modulation by SK channel block has not been reported in humans, but we ensured that our in-silico cardiomyocyte models reproduced the APD prolongation observed in patients with AF (Heijman et al., 2023).*

While this is calibration, not validation, we observed that our in-silico models also reproduced at tissue level the ERP prolongation reported for several animal models. This indeed counts as validation of the in-silico results, since, contrary to the APD, we did not fit the models to reproduce the ERP during calibration.

6. **Please specify the formulation used for ISK. Does this include a function describing the calcium dependent activation as the one optimised in [https://doi.org/10.1161/CIRCRESAHA.122.321858]?**

The formulations used for I_{SK} and I_{K2P} are explicitly described below and can be found in the papers that we cite. As the reviewer mentions, the I_{SK} activation is dependent on the intracellular calcium concentration, similar to the one described by Heijman *et al.*, 2023.

The baseline Courtemanche *et al.*, 1998 model was modified to include the K_2P channel family, as in Wiedmann *et al.*, 2020 (10.1161/JAHA.119.015751), and the SK channel, as in Celotto *et al.*, 2023 (10.1016/j.compbiomed.2023.106719). Moreover, the maximal conductances of I_{SK} and I_{K2P} were scaled by 1.1 and 1.7, respectively, to reproduce the 12% and 18% APD prolongation observed after single SK and K_2P channel block in cardiomyocytes from control patients (Heijman *et al.*, 2023).

The resulting ionic current formulations for the K_2P channel family is described in Equations 1–5:

$$p_{a(\infty)} = 0.2 + \frac{0.8}{1 + e^{-\frac{V_m+5}{14}}} \tag{1}$$

$$\tau_{p(a)} = 2.0 + \frac{40}{1 + e^{\frac{(V_m+25)^2}{80}}} \tag{2}$$

$$\frac{dp_a}{dt} = \frac{p_{a(\infty)} - p_a}{\tau_{p(a)}} \tag{3}$$

$$g_{K2P} = 1.7 * G_{K2P} * 0.0035 \tag{4}$$

$$I_{K2P} = g_{K2P} * p_a * (V_m - E_K) \tag{5}$$

where V_m is the transmembrane voltage, E_K is the Nernst potential for potassium, and g_{K2P} is the maximal conductance for the K_2P channel family, which as mentioned above is scaled by 1.7, while G_{K2P} is the additional scaling factor used for implementing the population of models.

The resulting ionic current formulations for the SK channel family is described in Equations 6-9:

$$s_{a(\infty)} = \frac{\left(\frac{[Ca^{2+}]_i}{0.0025}\right)^2}{1 + \left(\frac{[Ca^{2+}]_i}{0.0025}\right)^2} \tag{6}$$

$$\tau_{s(a)} = 3.0 \tag{7}$$

$$\frac{ds_a}{dt} = \frac{s_{a(\infty)} - s_a}{\tau_{s(a)}} \tag{8}$$

$$I_{SK} = 1.1 * G_{SK} * s_a^2 * (V_m - E_K) \tag{9}$$

where G_{SK} is the scaling factor resulting from the population of models approach, which is further multiplied by 1.1, as described above.

7. How many models were filtered with the first database (AP morphology) and how many with the second (APD prolongation after ISK block)?

From the candidate population of 500 atrial cardiomyocyte models, 240 (48%) were retained after the first calibration step (AP morphology). From this calibrated population, 50 (21%) were finally selected for multi-scale simulations after considering the experimental effects of I_{SK} block.

The greater reduction in the number of cardiomyocyte models after applying the second calibration step is largely due to the sample size of the two experimental datasets used. The first dataset (AP morphology) is the largest dataset of atrial trabeculae, consisting of **469 action potential recordings** from right atrial trabeculae derived from **214 patients in sinus rhythm and 149 patients with chronic AF**. These data were obtained in different Hospital centres from Germany, and show high inter-subject variability in action potential biomarkers, as described in Sanchez *et al.*, 2014 (Table 1; DOI: 10.1371/journal.pone.0105897.t001). Using this dataset for conducting a first calibration step allows retaining a larger number of in-silico atrial cardiomyocyte models, presenting great variability.

The second dataset consists of isolated cells from **six patients with chronic AF**. While this dataset covers some variability, the reduced sample size causes the calibrated population of models to shrink considerably in number. However, this is one of the few reliable datasets obtained in human samples treated with SK channel blockers.

8. Please include the reference for line 121 (volume rage observed in persistent AF patients).

Added, new **line 139 – 140**: *[…] spanning the volume ranges observed in persistent AF patients (Lang et al., 2015).*

9. I am concerned with the statement in line 135: "AF lasting longer than 7 s was considered sustained, since we previously showed in a similar cohort that all AF episodes sustaining over 7 s would also sustain for 30 s (i.e., duration used clinically for AF diagnosis)". I am not sure about this statement for the cases with smaller atrial dimensions. Can the authors demonstrate this and provide examples for the extreme scenarios?

We appreciate the reviewer's comment. To demonstrate that simulated AF sustained originally for 7 seconds would also sustain for 30 seconds even in extreme scenarios, we provide now results for the smallest atrial anatomy considered in our study (right and left atrial volumes of 64 and 59 mL, respectively), as requested. To further consider the more extreme scenarios, simulation results are presented in the absence of fibrotic remodelling (i.e., structurally-healthy atria), and considering the three atrial cardiomyocyte models with the longest refractoriness that presented sustained AF for 7 seconds.

These results complement the previous examples of sustained arrhythmia over 30 seconds which are still presented in **Figure 1** (showing one case of an unstable rotor in the crista terminalis of the right atrium and two cases of AF):

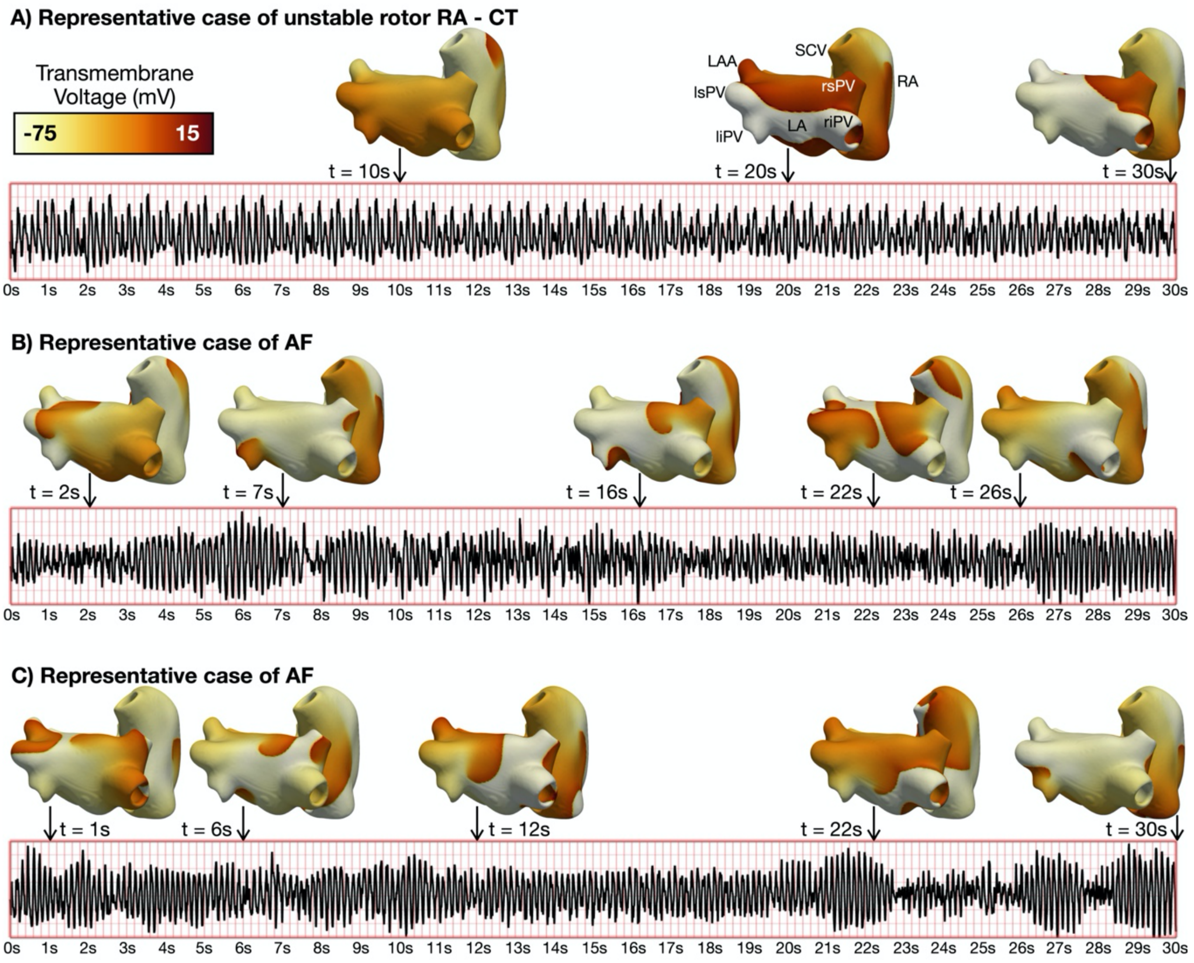

Figure S1. Examples of simulated atrial arrhythmia over 30 seconds. Snapshots of the atrial transmembrane voltage and corresponding ECG (lead II, no units) for a representative case of: **A)** unstable rotor in the crista terminalis (CT) of the right atrium (RA) and **B-C)** two cases of AF. **Abbreviations:** RA-LA: right and left atrium; LAA: LA appendage; SCV: superior cava vein: rs-ri-ls-li-PV: right superior, right inferior, left superior and left inferior pulmonary vein.

10. Line 384: the authors state that "non-cardioverted virtual patients presented stable rotors in the atria even after drug application, which remained unmodified during the entire duration of the AF episode". Could the authors be more specific (i.e., is the dominant frequency checked over time, number of rotors, etc)?

We thank the reviewer for their comment. The number and location of rotors are checked over time. **Figure S2** illustrates an example in which stable rotors in the left atrial posterior wall can be seen in control conditions and after simulating two pharmacological interventions, SK and K_2P channel block:

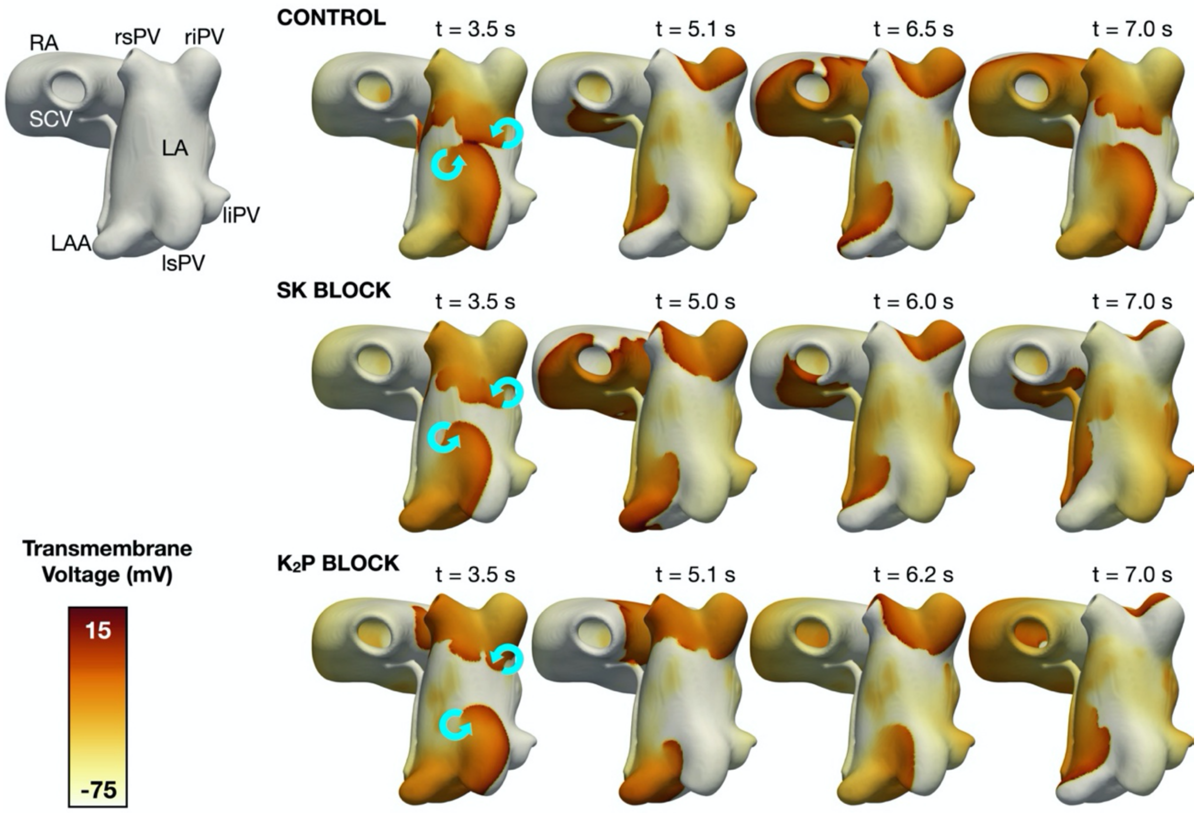

Figure S2. Snapshots of the atrial transmembrane voltage illustrating stable rotors in the left atrial posterior wall in control conditions and after two pharmacological interventions, SK and K_2P channel block, throughout the entire duration of the simulation. **Top-left)** Schematic representation of the biatrial model. **Abbreviations:** RA-LA: right and left atrium; LAA: LA appendage; SCV: superior cava vein: rs-ri-ls-li-PV: right superior, right inferior, left superior and left inferior pulmonary vein.

This has been clarified in the main article:

Line 437 – 439: *Accordingly, non-cardioverted virtual patients presented stable rotors in the atria even after drug application, which remained unmodified during the entire duration of the AF episode (i.e., similar number and location of rotors over time).*

Minor comments: The sentence in line 187 is repetitive with the previous paragraph.

Modified, new **line 206 – 208**: *While a similar efficacy derived from single SK and K2P channel inhibition, from the 213 virtual patients responding to I_{SK} block and the 278 ones responding to I_{K2P} block, only 108 virtual patients were cardioverted by both treatments.*

Dear Dr Dasí,

Re: JP-RP-2024-287124R1 "Prospective in-silico trials identify combined SK and K2P channel block as an effective strategy for atrial fibrillation cardioversion" by Albert Dasí, Lucas Arantes Berg, Hector Martinez-Navarro, Alfonso Bueno Orovio, and Blanca Rodriguez

Thank you for submitting your manuscript to The Journal of Physiology. It has been assessed by a Reviewing Editor and by 2 expert referees and we are pleased to tell you that it is potentially acceptable for publication following satisfactory major revision.

Please advise your co-authors of this decision as soon as possible.

The referee reports are copied at the end of this email.

Please address all the points raised and incorporate all requested revisions or explain in your Response to Referees why a change has not been made. We hope you will find the comments helpful and that you will be able to return your revised manuscript within 2 months. If you require longer than this, please contact journal staff: jp@physoc.org. Please note that this letter does not constitute a guarantee for acceptance of your revised manuscript.

Your revised manuscript should be submitted online using the link in your Author Tasks: Link Not Available. This link is accessible via your account as Corresponding Author; it is not available to your co-authors. If this presents a problem, please contact journal staff (jp@physoc.org). Image files from the previous version are retained on the system. Please ensure you replace or remove any files that are being revised.

If you do not wish to submit a revised version of your manuscript, you must inform our journal staff (jp@physoc.org) or reply to this email to request withdrawal. Please note that a manuscript must be formally withdrawn from the peer review process at one journal before it may be submitted to another journal.

TRANSPARENT PEER REVIEW POLICY: To improve the transparency of its peer review process, The Journal of Physiology publishes online as supporting information the peer review history of all articles accepted for publication. Readers will have access to decision letters, including Editors' comments and referee reports, for each version of the manuscript, as well as any author responses to peer review comments. Referees can decide whether or not they wish to be named on the peer review history document.

ABSTRACT FIGURES: Authors are expected to use The Journal's premium BioRender account to create/redraw their Abstract Figures. Information on how to access this account is here: https://physoc.onlinelibrary.wiley.com/journal/14697793/biorender-access.

This will enable Authors to create and download high-resolution figures. If authors have used the free BioRender service, they can use the instructions provided in the link above to download a high-resolution version suitable for publication.

The link provided should only be used for the purposes of this submission. Authors will be charged for figures created on this account if they are not related to this manuscript submission.

LANGUAGE EDITING AND SUPPORT FOR PUBLICATION: If you would like help with English language editing, or other article preparation support, Wiley Editing Services offers expert help, including English Language Editing, as well as translation, manuscript formatting, and figure formatting at www.wileyauthors.com/eeo/preparation. You can also find resources for Preparing Your Article for general guidance about writing and preparing your manuscript at www.wileyauthors.com/eeo/prepresources.

RIGOUR AND REPRODUCIBILITY: We recommend authors consult The Journal's Rigour and Reproducibility requirements to ensure that their manuscript is suitable for publication: https://physoc.onlinelibrary.wiley.com/pb-assets/hub-assets/physoc/documents/TJP-Rigour-and-Reproducibility-Requirements.pdf

REVISION CHECKLIST:

Check that your Methods section conforms to journal policy: https://jp.msubmit.net/cgi-bin/main.plex?form_type=display_requirements#methods.

Check that data presented conforms to the statistics policy: https://jp.msubmit.net/cgi-bin/main.plex?form_type=display_requirements#statistics.

Upload a full Response to Referees file. To create your 'Response to Referees': copy all the reports, including any comments from the Senior and Reviewing Editors, into a Microsoft Word, or similar, file and respond to each point, using font or background colour to distinguish comments and responses and upload as the required file type.

Please upload two versions of your manuscript text: one with all relevant changes highlighted and one clean version with no changes tracked. The manuscript file should include all tables and figure legends, but each figure/graph should be uploaded as separate, high-resolution files.

You may also upload:

- 'Potential Cover Art' for consideration as the issue's cover image
- Appropriate Supporting Information (Video, audio or data set: see https://jp.msubmit.net/cgi-bin/main.plex?form_type=display_requirements#supp).

We look forward to receiving your revised submission.

If you have any queries, please reply to this email and we will be pleased to advise.

Yours sincerely,

Bjorn Knollmann
Senior Editor
The Journal of Physiology

EDITOR COMMENTS

Reviewing Editor:

I agree with referee 1 that the limitations section is contradictory. I acknowledge that very long simulation times are not feasible due to the complexity of the simulations, but recommend increasing the run time for (at least) one simulation.

Senior Editor:

While improved, there are still concerns remaining that will need to be addressed before the manuscript can be accepted.

REFEREE COMMENTS

Referee #1:

The authors have revised the manuscript and addressed the points that have been raised. I appreciate the extension of Discussion regarding the possible (in)direct effects of ISK inhibition on INa. After carefully reviewing the manuscript again, I have one major and a few minor suggestions:

Major suggestion:

One of the possible explanations for lower cardioversion efficacy of simulated pharmacological interventions when compared to published clinical trials is the very short observation period (5s) after the intervention. In Limitations (lines 442-443), the authors claim that "longer simulation times would unlikely change the outcomes of the study", yet, at the beginning of the same paragraph they state that "it is unclear whether 7s of simulated AF is equivalent to the 90 minutes waiting reported in the trials" (lines 429-430). To support their claim about the observation period of 5s being sufficient, I think the authors should consider to run the simulations in (at least some) non-cardioverting patients for longer, to demonstrate (or at least illustrate) that the study outcomes would not change if the simulation times were longer.

Minor suggestions:

1) The authors should provide a description on how ERP was measured. I have not found this description anywhere in the manuscript, yet ERP data are reported in Figure 4.

2) Figure 2A: My previous comment regarding the word "respectively" referred to Figure 2A heading (262 and 392 with absence and presence of LVA, "respectively"), not to the figure description.

3) Line 322: There is a typo, the compound's name is AP14145.

4) Lines 423-425: The sentence is difficult to understand.

Referee #2:

The authors have addressed all the points, and I have no further comments.

END OF COMMENTS

1st Confidential Review **18-Sep-2024**

EDITOR COMMENTS

Reviewing Editor

I agree with referee 1 that the limitations section is contradictory. I acknowledge that very long simulation times are not feasible due to the complexity of the simulations, but recommend increasing the run time for (at least) one simulation.

Senior Editor

While improved, there are still concerns remaining that will need to be addressed before the manuscript can be accepted.

We appreciate the time and effort from the editors and reviewers. The manuscript has been newly revised considering the reviewer' additional comments. Ten simulations of pharmacological treatment, each with a different atrial anatomy, have been run for a longer duration of 12 seconds.

REFEREE COMMENTS

Referee #1:

The authors have revised the manuscript and addressed the points that have been raised. I appreciate the extension of Discussion regarding the possible (in)direct effects of ISK inhibition on INa.

We thank the reviewer for their previous comment. We agree that the potential effects of SK channel inhibition on I_{Na} were worth discussing.

After carefully reviewing the manuscript again, I have one major and a few minor suggestions:

Major suggestion:

- **One of the possible explanations for lower cardioversion efficacy of simulated pharmacological interventions when compared to published clinical trials is the very short observation period (5s) after the intervention. In Limitations (lines 442-443), the authors claim that "longer simulation times would unlikely change the outcomes of the study", yet, at the beginning of the same paragraph they state that "it is unclear whether 7s of simulated AF is equivalent to the 90 minutes waiting reported in the trials" (lines 429-430). To support their claim about the observation period of 5s being sufficient, I think the authors should consider to run the simulations in (at least some) non-cardioverting patients for longer, to demonstrate (or at least illustrate) that the study outcomes would not change if the simulation times were longer.**

  We appreciate the reviewer's concern. We have conducted new simulations in a representative cohort of 10 non-cardioverted virtual patients that originally presented sustained AF for 5 seconds after drug simulation. The new simulations were run for 5 additional seconds, for a total duration of 10 seconds of simulated pharmacological action. The two pharmacological treatments resulting in lower cardioversion efficacy (i.e., single SK and K2P channel block) were considered. **Figure S1** shows sustained AF in these 10 virtual patients, each of them presenting a different atrial anatomy and electrophysiological properties. It can be appreciated that the longer simulation time does not result in AF cardioversion or in a reduction of arrhythmia complexity. (Figure S1 can be found in this document after the Reviewer's minor comments).

  This is in line with what we already mentioned in the Limitations section, and in our previous comment to **Referee #2**, that non-cardioverted virtual patients presented stable rotors in the atria even after drug application, which remained unmodified during the entire duration of the AF episode (**Figure S2**).

  We have rephrased the first assertion in the Limitations section ("*it is unclear whether 7s of simulated AF is equivalent to the 90 minutes waiting reported in the trials* "), added a sentence in the discussion to reflect the new results and made the longer simulations available at ZENODO, together with a <u>unified Supplemental Material that collects the discussion with the Referees</u>: (https://zenodo.org/records/13907120).

Line 437: *However, it is difficult to anticipate whether 7s of simulated AF is equivalent to the 90 minutes waiting reported in the trials.*

Line 451 – 454: *The significant difference in ERP prolongation between cardioverted vs. non-cardioverted virtual patients and the stable AF dynamics seen in non-cardioverted patients after simulated drug treatment is reassuring that longer simulation times would unlikely change the outcomes of the study. Indeed, in a representative cohort of 10 virtual patients with variability in anatomy and electrophysiology that were not cardioverted after 5 s of simulated drug action, a longer simulation time of 10 seconds did not result in AF cardioversion or in a reduction of arrhythmia complexity. These simulations are available at https://zenodo.org/records/13907120.*

Moreover, a reference to these simulations can be found in the Methods section:

Line 165 – 167: *Longer simulations of 12 s were conducted in a subset of 10 virtual patients to ensure that the cardioversion efficacy and arrhythmia dynamics were independent of the simulation time (results available at https://zenodo.org/records/13907120).*

Minor suggestions:

1. **The authors should provide a description on how ERP was measured. I have not found this description anywhere in the manuscript, yet ERP data are reported in Figure 4.**

   A description of how the ERP was calculated is now included in the main manuscript.

   Line 173 – 178: *The effective refractory period (ERP) was calculated in control conditions and after drug simulation using a cable tissue of 5.00×0.04×0.04 cm^3, with a spatial discretization of 400 µm (same as in multi-scale simulations). Five consecutive beats were paced at one side of the cable at a constant pacing rate, each time with decreasing cycle lengths. Cardiomyocyte models were previously paced for 50 beats in a single-cell environment, so they could adapt to the different cycle lengths. The ERP was measured as the shortest cycle length at which all five beats propagated through the cable.*

2. **Figure 2A: My previous comment regarding the word "respectively" referred to Figure 2A heading (262 and 392 with absence and presence of LVA, "respectively"), not to the figure description.**

   This has been changed. New Figure 2.

3. **Line 322: There is a typo, the compound's name is AP14145.**

   The compound's name has been corrected.

4. **Lines 423-425: The sentence is difficult to understand**

   The sentence has been rephrased.

   Line 431 – 433: *Thus, considering sex-specific differences could be critical for planning pharmacological treatment. However, human data, especially from the atria, are needed to confirm gender differences in SK and K2P channels.*

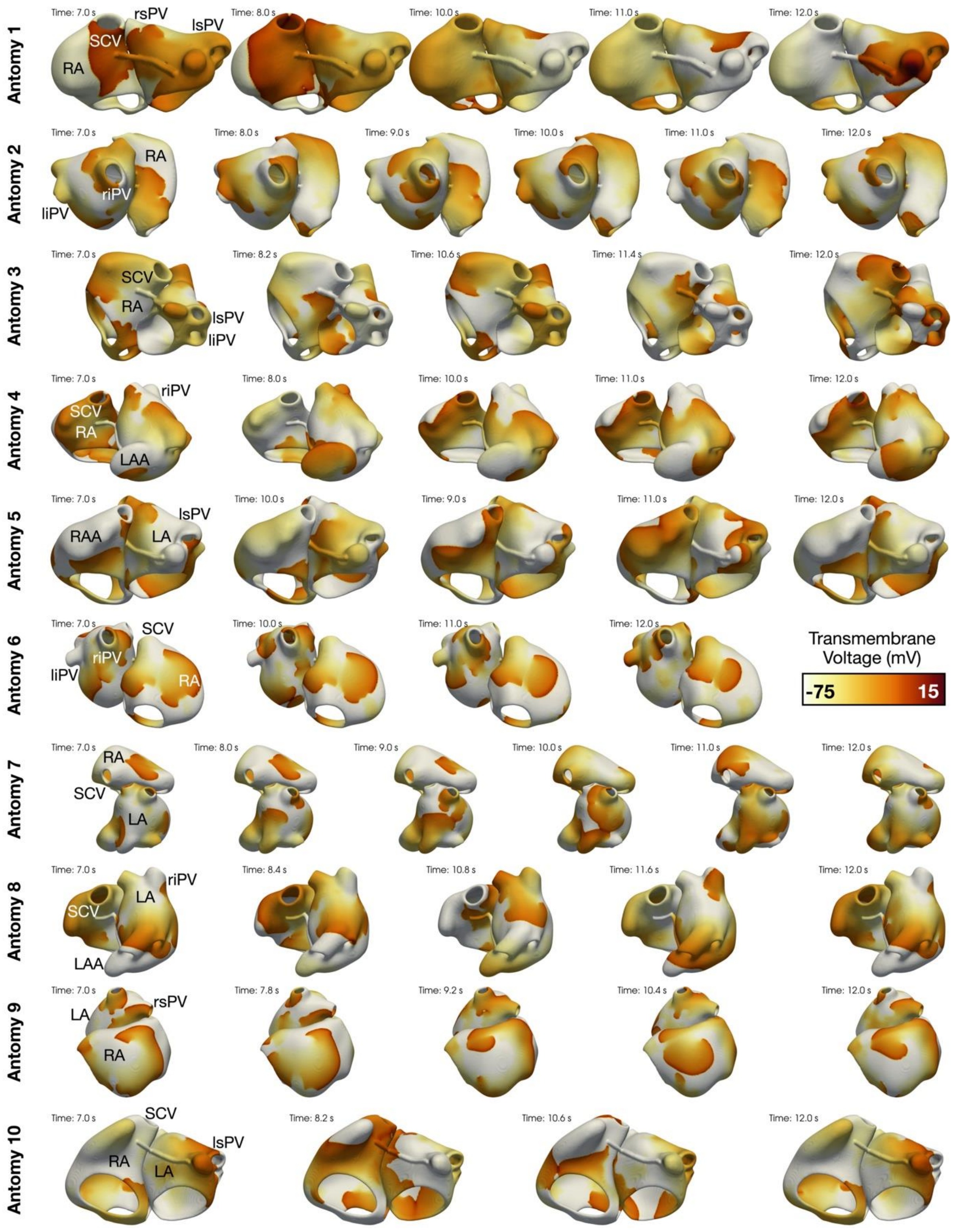

Figure S1. Consecutive snapshots of the atrial transmembrane voltage in 10 virtual patients with sustained AF despite pharmacological treatment. The first five virtual patients (i.e., anatomy 1 – 5) presented sustained AF after SK channel block and the following five (i.e., anatomy 6 – 10) after K_2P channel block. Pharmacological treatment is simulated for 10 seconds after 2 seconds from AF initiation, for a total simulation time of 12 seconds. **Abbreviations:** RA-LA: right and left atrium; RAA-LAA: RA and LA appendage; SCV: superior cava vein; rs-ri-ls-lr-PV: right superior, right inferior, left superior and left inferior pulmonary vein.

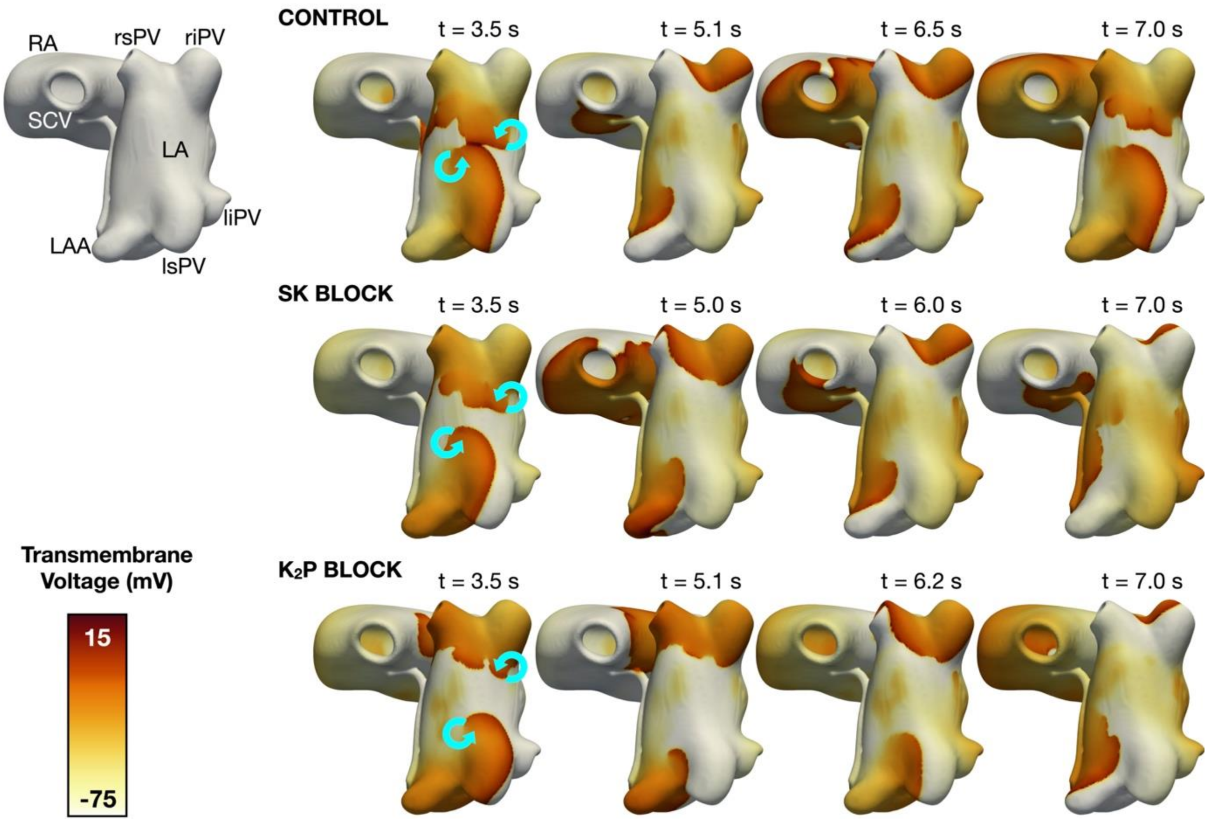

Figure S2. Consecutive snapshots of the atrial transmembrane voltage illustrating stable rotors in the left atrial posterior wall in control conditions and after two pharmacological interventions, SK and K_2P channel block, throughout the entire duration of the simulation. **Top-left)** Schematic representation of the biatrial model. **Abbreviations:** RA-LA: right and left atrium; LAA: LA appendage; SCV: superior cava vein: rs-ri-ls-li-PV: right superior, right inferior, left superior and left inferior pulmonary vein.

Dear Mr Dasí,

Re: JP-RP-2024-287124R2 "Prospective in-silico trials identify combined SK and K2P channel block as an effective strategy for atrial fibrillation cardioversion" by Albert Dasí, Lucas Arantes Berg, Hector Martinez-Navarro, Alfonso Bueno Orovio, and Blanca Rodriguez

We are pleased to tell you that your paper has been accepted for publication in The Journal of Physiology.

The last Word (or similar) version of the manuscript provided will be used by the Production Editor to prepare your proof. When this is ready you will receive an email containing a link to Wiley's Online Proofing System. The proof should be thoroughly checked and corrected as promptly as possible.

Authors should note that it is too late at this point to offer corrections prior to proofreading. Major corrections at proof stage, such as changes to figures, will be referred to the Editors for approval before they can be incorporated. Only minor changes, such as to style and consistency, should be made at proof stage. Changes that need to be made after proof stage will usually require a formal correction notice.
All queries at proof stage should be sent to: TJP@wiley.com.

Following copyediting and typesetting the accepted version will be published online.

Yours sincerely,

Bjorn Knollmann
Senior Editor
The Journal of Physiology

IMPORTANT POINTS TO NOTE FOLLOWING YOUR PAPER'S ACCEPTANCE:

If you would like to receive our 'Research Roundup', a monthly newsletter highlighting the cutting-edge research published in The Physiological Society's family of journals (The Journal of Physiology, Experimental Physiology, Physiological Reports, The Journal of Nutritional Physiology and The Journal of Precision Medicine: Health and Disease), please click this link, fill in your name and email address and select 'Research Roundup':
https://www.physoc.org/journals-and-media/membernews

• TRANSPARENT PEER REVIEW POLICY: To improve the transparency of its peer review process, The Journal of Physiology publishes online as supporting information the peer review history of all articles accepted for publication. Readers will have access to decision letters, including Editors' comments and referee reports, for each version of the manuscript as well as any author responses to peer review comments. Referees can decide whether or not they wish to be named on the peer review history document.

• You can help your research get the attention it deserves! Check out Wiley's free Promotion Guide for best-practice recommendations for promoting your work at: www.wileyauthors.com/eeo/guide. You can learn more about Wiley Editing Services which offers professional video, design, and writing services to create shareable video abstracts, infographics, conference posters, lay summaries, and research news stories for your research at: www.wileyauthors.com/eeo/promotion.

• IMPORTANT NOTICE ABOUT OPEN ACCESS: To assist authors whose funding agencies mandate public access to published research findings sooner than 12 months after publication, The Journal of Physiology allows authors to pay an Open Access (OA) fee to have their papers made freely available immediately on publication.

The Corresponding Author will receive an email from Wiley with details on how to register or log-in to Wiley Authors Services where you will be able to place an order

• You can check if your funder or institution has a Wiley Open Access Account here: https://authorservices.wiley.com/author-resources/Journal-Authors/licensing-and-open-access/open-access/author-compliance-tool.html.

EDITOR COMMENTS

Comments to the Author:
Thank you for the nicely revised manuscript.

Senior Editor:

Comments to the Author:
The MS is now acceptable for publication. Excellent in silico work with significant translational implications!

REFEREE COMMENTS

Referee #1:

I thank the authors for addressing my comments. I do not have any additional suggestions.

2nd Confidential Review **14-Oct-2024**
